# Single-cell sequencing and organoids: applications in organ development and disease

**DOI:** 10.1186/s43556-025-00364-6

**Published:** 2025-12-08

**Authors:** Tiantian Li, Jianmei Yin, Yang Hao, Wei Gao, Qirong Li, Qiang Feng, Boqiang Tao, Ming Hao, Yanxin Liu, Chao Lin, Dongxu Wang

**Affiliations:** 1 Laboratory Animal Center, College of Animal Science, Jilin University, Changchun, 130021 China; 2 Department of Otorhinolaryngology, The First Hospital of Jilin University, Changchun, China; 3 Department of Oral and Maxillofacial Surgery, Hospital of Stomatology, Jilin University, Changchun, China; 4 Department of Gastric and Colorectal Surgery, General Surgery Center, The First Hospital of Jilin University, Changchun, China; 5 School of Grain Science and Technology, Jilin Business and Technology College, Changchun, China; 6 Zhichuang Gene Editing Animal Model Research Centre, Health for All Research Centre, Wenzhou Institute of Technology, Wenzhou, China

**Keywords:** Single-cell sequencing, Organoids, Development, Disease modeling, Cancer research

## Abstract

The integration of single-cell sequencing and organoid technologies has been transformative for biomedical research, enabling investigations of organ development, disease mechanisms, and therapeutic innovation at even finer resolutions. Organoids serve as 3D in vitro models that replicate the structural and functional complexity of human tissues, while single-cell sequencing can resolve cellular heterogeneity, transcriptional dynamics, and lineage trajectories at high resolution. This review systematically explores the synergistic potential of these two technologies across multiple domains. First, it describes their application in studying the developmental mechanisms of organs including the brain, lungs, heart, liver, intestines, and kidneys, revealing key signaling pathways and cellular interaction networks. Then, it details their application in studying in vitro models of various diseases, including neurodegenerative disorders, genetic diseases, infectious diseases, metabolic syndrome, and tumors, advancing the in-depth analysis of pathological mechanisms. By leveraging patient-derived organoid biobanks, combining these two technologies can accelerate drug screening and precision, while utilizing transplantable tissue constructs to pioneer regenerative medicine strategies. This review also highlights the strengths of combining these two technologies in dynamically decoding cellular behavior and communication networks. By constructing physiologically relevant multifunctional research platforms, the integration of single-cell sequencing with organoid models will accelerate the elucidation of disease mechanisms and drive innovative breakthroughs in precision medicine and regenerative medicine. Looking ahead, the deep integration of single-cell sequencing with organoids, combined with cutting-edge technologies such as spatial transcriptomics and gene editing, will continue to propel life sciences toward a transformative leap from descriptive research to mechanism-driven, precision-oriented, and personalized approaches.

## Introduction

Biological research was long confined to the two-dimensional (2D) cell culture world, where cells are examined in an environment lacking their natural three-dimensional (3D) structure and complex intercellular communication, making it challenging to recapitulate the complexity of biological activities in a living organism [1, 2]. The advent of organoids has completely overcome this limitation. Organoids are 3D multicellular culture systems that provide biomechanical support and biochemical signals. They are the best in vitro model of cellular heterogeneity because they accurately mimic the complex interactions present in human physiology [3]. Their cultivation typically requires an artificial extracellular connection between cells and their surrounding environment [4]. This unique culture environment enables organoids to replicate the intricate physiological structures of natural tissues or organs, offering a more precise reflection of the in vivo environment compared to traditional 2D cell cultures [5, 6]. Consequently, organoids offer a superior platform for studying the mechanisms involved in organ development and disease pathogenesis, as well as screening drugs. Organoids can be generated from diverse sources, including individuals with different genetic backgrounds, as well as from adult stem cells (ASCs), embryonic stem cells (ESCs), or induced pluripotent stem cells (iPSCs) [7]. Recent studies have successfully engineered organoids from diverse tissues, including renal tubules, fetal colons, and various brain regions, enabling the recapitulation of dynamic processes involved in organ development and disease in culture dishes. This advancement has elevated in vitro research to new heights [2, 8].

While organoids provide a miniature landscape for observing biological processes, single-cell sequencing acts as a microscope, distinguishing changes within this microscopic landscape [9]. In modern biomedical research, there is an urgent need to leverage advanced technologies to comprehensively analyze the complex and diverse mechanisms underlying disease pathogenesis. These efforts aim to elucidate the core pathological characteristics and identify key pathogenic pathways, providing a robust scientific foundation for advancing precision and personalized medicine [10]. Traditional sequencing methods often involve the collective analysis of large numbers of cells, capturing only the average characteristics of cell populations, and are unable to reveal intercellular variability [11]. In contrast, next-generation single-cell sequencing enables the precise examination of the genome, transcriptome, and epigenome at the single-cell level, which is vital for detailed analysis of cell heterogeneity [12, 13]. Cell heterogeneity exists widely in various biological systems. In tissues or organs, cells of the same type exhibit differences in gene expression, metabolic state, cell function, and cell cycle [14, 15]. Single-cell sequencing can precisely dissect these differences, providing a comprehensive and in-depth understanding of the microscopic mechanisms of disease development [16].

**Fig. 1:**
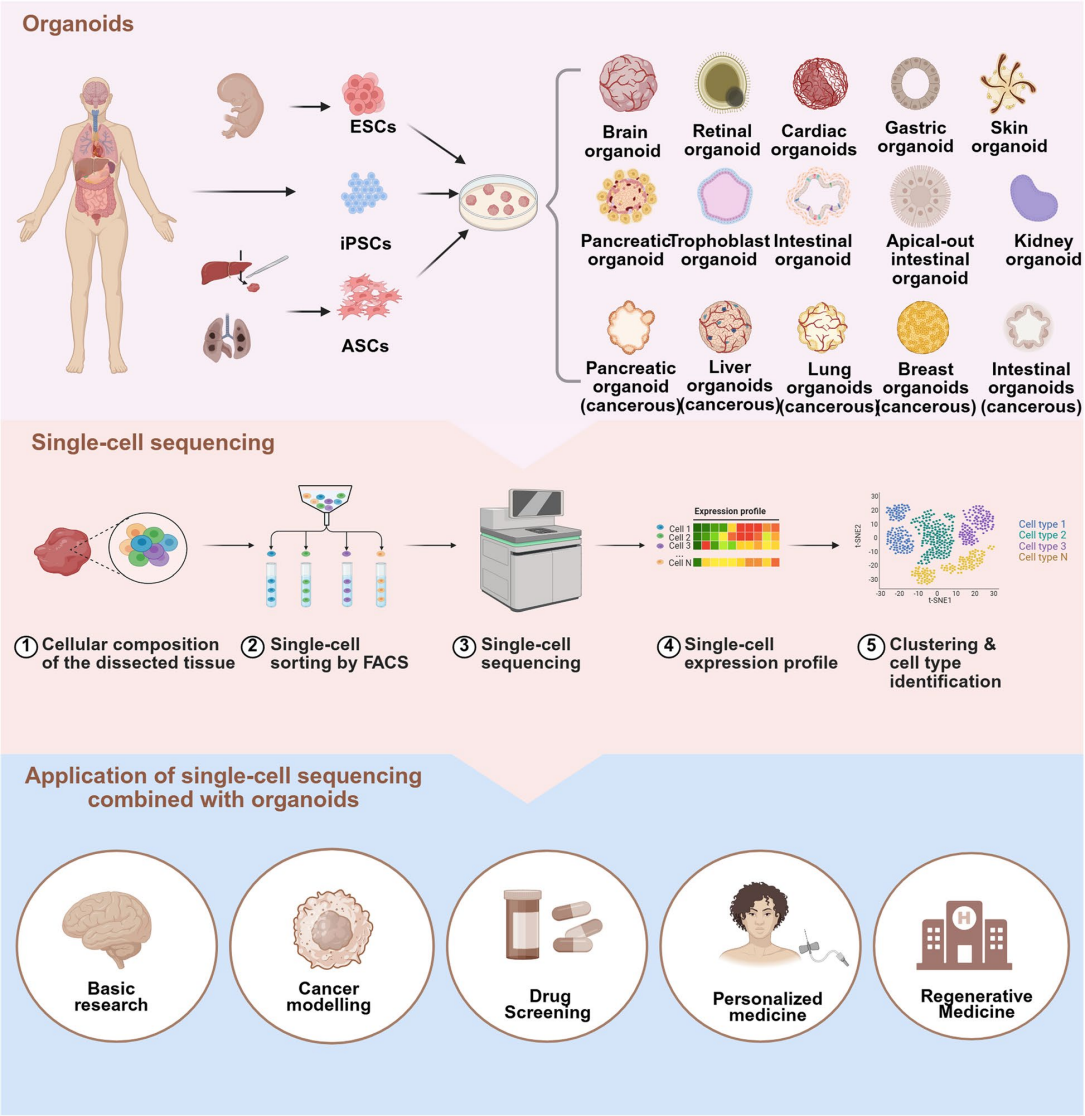
The Coapplication of Organoid and Single-Cell Sequencing Technologies. Organoids are 3D structures that replicate specific organ development, structure, and function, exhibiting high biological fidelity in vitro. They can be derived from ESCs, iPSCs, or ASCs from normal or tumor tissues, enabling the generation of diverse organs (e.g., liver, kidney, and brain) and tumor organoids. Single-cell sequencing is a key tool in modern life sciences. Its workflow involves dissociating target tissues or organoids into single-cell suspensions, sorting cells via fluorescence-activated cell sorting (FACS) or microfluidics, and performing high-throughput sequencing to obtain gene expression profiles. It enables the identification of cell types, subpopulations, and their functional heterogeneity through cluster analysis. Combinating organoids and single-cell sequencing has driven innovations across research fields. Organoids provide platforms for studying organ development, disease modeling, and gene editing. The single-cell sequencing of patient-derived tumor organoids can facilitate drug screening, resistance studies, and personalized medicine. Organoids also hold potential in regenerative medicine. This integration enhances research precision, accelerates biomedical advancements, and offers new opportunities for disease prevention, drug development, and therapeutic innovation

Combining single-cell sequencing with organoids offers a novel perspective for exploring various unknowns in human diseases. It also offers new opportunities to gain a deeper understanding of the mechanisms underlying cell behavior and lineage differentiation during normal organ development [17]. Organoids can be used as miniature in vitro models of developmental processes to reproduce key events in organogenesis [2]. Single-cell multi-omics technologies can accurately capture the dynamic changes in cell state during this process, revealing the temporal and spatial order of gene expression and epigenetic regulation, thus systematically elucidating the molecular basis of normal development [18]. In cancer research, integrating single-cell sequencing with tumor organoids can facilitate the delineation of cellular heterogeneity within tumors, the identification of cancer stem cells, and the characterization of different subclones, providing a basis for precise diagnosis and personalized treatment [19]. In drug screening, organoids serve as efficient platforms, while single-cell sequencing can monitor cellular responses to drugs to assess their efficacy and toxicity [20]. In regenerative medicine, the integration of organoids with single-cell sequencing presents considerable promise, providing robust theoretical foundations for advancing tissue repair and regeneration methodologies [21, 22]. Thus, combining organoids with single-cell sequencing can advance our understanding of disease mechanisms and normal development, serving as a powerful tool for modern biomedical research and clinical practice (Fig. 1).

Therefore, this review aims to systematically trace the development history and cutting-edge advancements in integrating single-cell sequencing and organoids. It first outlines the delicate developmental blueprints of organs revealed by this combined approach. It then examines its breakthrough applications in disease modeling, tumor heterogeneity analysis, drug screening, and regenerative medicine. It will also objectively analyze the challenges and limitations currently facing this technological integration and thereby explore its potential future development directions. Ultimately, this review will highlight the potential of joint analysis using organoids and single-cell sequencing to provide new insights into cellular behavior and signal transduction mechanisms, as well as the potential for further innovation and breakthroughs in biomedical research.

## Overview of organoids and single-cell sequencing

### Establishment and characterization of an organoid culture system

**Fig. 2:**
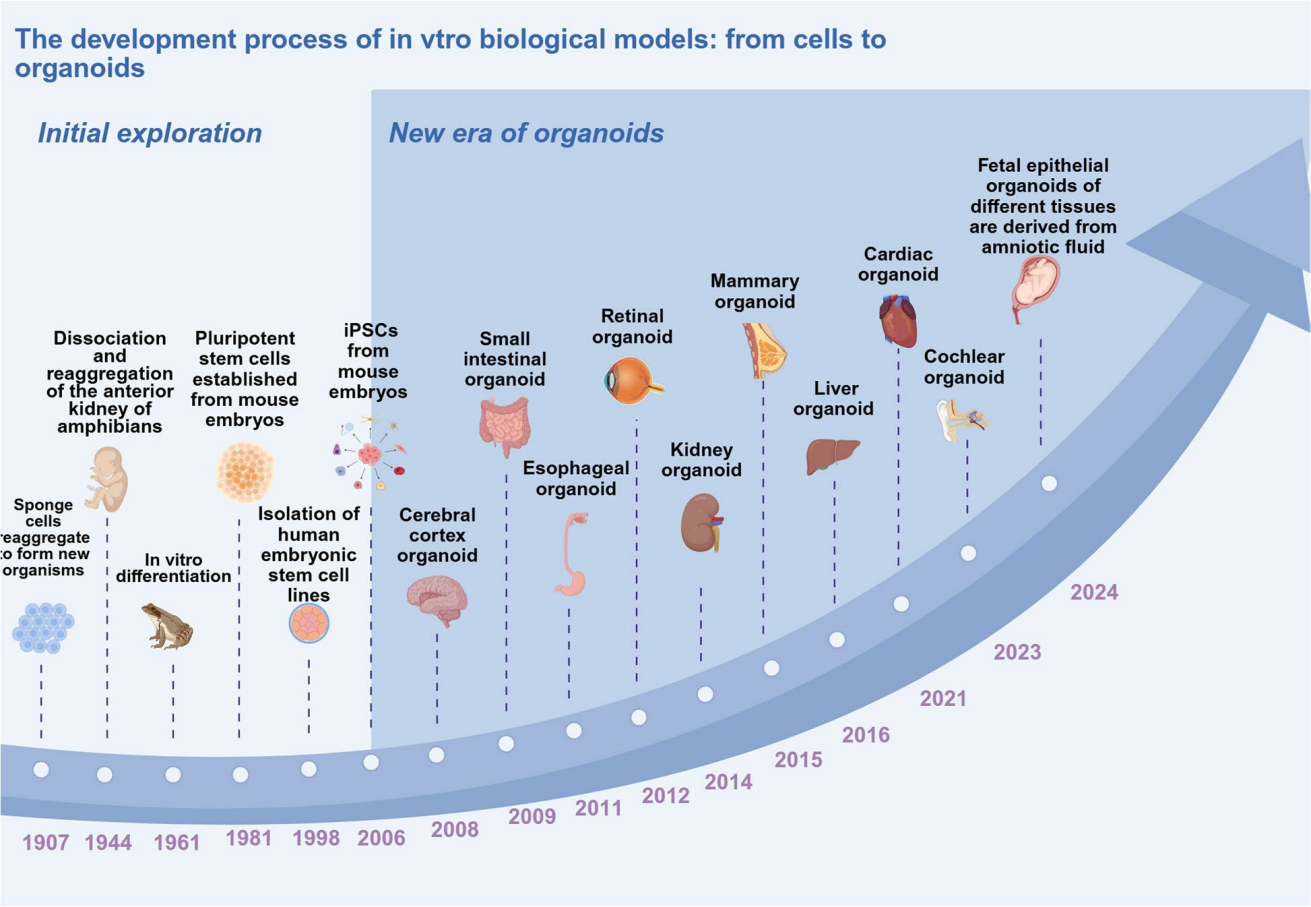
The Developmental History of Organoid Technology. Organoid technology developed over a century, beginning with the discovery of sponge cells reaggregating to form new organisms in 1907. The dissociation and reaggregation of amphibian pronephros revealed cells’ self-organizing capability in 1944. The in vitro differentiation of embryoid bodies further demonstrated cellular potential in 1961. The establishment of pluripotent stem cells from mouse embryos in 1981, followed by the successful isolation of human ESCs (hESCs) in 1998, laid the foundation for organoid research. The reprogramming of mouse somatic cells led to the creation of iPSCs in 2006. Since 2008, researchers have developed various organoids, including those of the cerebral cortex, small intestine, esophagus, retina, kidney, mammary gland, liver, heart, and cochlea, providing crucial models for disease research and drug screening. By 2024, fetal epithelial organoids from diverse tissues were derived from amniotic cells. Organoid technology not only reveals the enigmas of life’s self-organization but also drives advancements in regenerative medicine and personalized therapy, offering unlimited potential for human health

Since the first successful cultivation of small intestinal organoids with crypt-villus structures in 2009 [23], organoids have marked a foundational breakthrough in biomedical research. Through them, ASCs were shown to self-assemble in a 3D environment in vitro, which provided a theoretical basis and technical paradigm for the subsequent establishment of multi-tissue organoids [3]. The rapid advancement in organoids has led to rapid growth in organoid research. Multilayered organoids have been successively established, ranging from digestive and respiratory organs, including the intestine, liver, and lung, derived from the endoderm, to circulatory and excretory systems, including the kidney, heart, and blood vessels, derived from the mesoderm, and extending to neural sensory tissues, including the brain and retina, derived from the ectoderm (Fig. 2) [8, 24–27]. These groundbreaking achievements fully demonstrate the immense potential of stem cells for self-organization in 3D space, propelling organoids from basic research towards clinical application [28–34].

Recently, organoids have exhibited a multidimensional trend in innovative development [4, 35]. The introduction of gene editing technologies has significantly enhanced the precision modeling capabilities of organoid systems [36]. Technologies such as clustered regularly interspaced short palindromic repeats (CRISPR) and base editing enable the precise introduction of specific genetic mutations into organoids, simulating the onset and progression of monogenic hereditary diseases, as well as complex diseases [37, 38]. Additionally, innovations in engineered culture systems have significantly expanded the application scope of organoids [39]. The establishment of multi-organ co-culture systems has enabled the synergistic cultivation of organoids from different organs, simulating physiological interactions between organs [40]. Vascularized organoids partially address limitations in nutrient delivery and metabolic waste removal within traditional organoids by introducing endothelial cells (ECs), thereby enhancing the survival time and functional maturity of organoids [41].

Compared to traditional research models, organoids exhibit unique advantageous characteristics [42]. Unlike 2D cell cultures, the 3D structure of organoids can more authentically recapitulate cellular interactions, establishment of cell polarity, and formation of tissue structures within the in vivo microenvironment, providing an ideal platform for studying cell–cell and cell–matrix interactions. Compared to animal models, organoids offer numerous advantages, including lower costs, shorter research cycles, and fewer ethical restrictions [43, 44]. Notably, through the use of patient-derived iPSCs, they can circumvent species differences to achieve personalized modeling. Furthermore, organoids demonstrate exceptional scalability, enabling their use in high-throughput drug screening and toxicity testing, significantly enhancing the efficiency of drug development [2].

Organoids have become an ideal in vitro platform for studying organ development, disease mechanisms, and drug screening. With the continuous optimization and innovation of organoid culture systems, their application prospects within precision medicine, regenerative medicine, and toxicological evaluation will become even broader. In the future, greater breakthroughs are anticipated in simulating complex physiological and pathological processes, as well as enabling long-term dynamic monitoring through the deep integration of cutting-edge technologies, including single-cell sequencing, microfluidic chips, and organoids.

### Evolution and integration of single-cell sequencing

The introduction of single-cell sequencing has enabled the high-throughput sequence analysis of the genome, transcriptome, or epigenome of single cells. Its core advantage lies in its ability to uncover the heterogeneity masked by traditional bulk sequencing within cell populations [45, 46]. Next-generation sequencing has progressed from population-level to single-cell resolution, and subsequently to the integration of multi-omics and spatial localization. The origins of single-cell sequencing can be traced back to early exploratory studies on individual cells [47]. With the gradual advancement of molecular biology techniques and the increasing demand for research on cellular heterogeneity [48–52], single-cell sequencing technologies began to emerge [53–55]. The first single-cell transcriptome sequencing method was proposed in 2009. Since then, the technology has progressively evolved, from the first-generation, low-throughput, microwell plate-based methods to the current high-throughput, multi-omics integrated, and spatially resolved methods [56].

In the initial phase, bulk next-generation RNA sequencing (RNA-seq) laid the bioinformatics foundation for subsequent technologies by extracting and sequencing mixed RNA from a large number of cells, providing population-averaged gene expression profiles, but completely masking cellular heterogeneity [56–59]. Single-cell RNA sequencing subsequently overcame this limitation, enabling the analysis of gene expression at the single-cell level. In 2011, single-cell tagged reverse transcription sequencing (STRT-seq) introduced cell barcoding, which supports the large-scale detection of various mixed cell samples, especially those with high heterogeneity [60, 61]. Switching mechanism at the 5’ end of RNA template sequencing (SMART-seq) and the subsequent SMART-seq2 have improved the full-length transcriptome coverage and sensitivity of single-cell sequencing, addressing the challenges of whole-genome transcriptome analysis in rare cells [62–64]. Furthermore, cell expression by linear amplification and sequencing (CEL-seq) enables multiplex analysis and study of different single cells [65].

With technological advancements and refinements, single-cell sequencing has entered the era of high throughput and automation. Droplet sequencing (Drop-seq) integrates microfluidics with single-cell sequencing, enabling the simultaneous processing of tens of thousands of single cells [66, 67]. This technology achieved a qualitative breakthrough in cell throughput, making automated single-cell transcriptomics possible and marking the end of the era of manual single-cell transcriptome sequencing [66]. In 2015, microfluidic chips were utilized to isolate individual cells and perform genomic sequencing, enabling more efficient single-cell sequencing. Then, in 2017, cellular indexing of transcriptomes and epitopes by sequencing (CITE-seq) was introduced, which integrates single-cell sequencing with the identification of protein epitopes. This technology enables simultaneous detection of transcriptome and surface protein expression levels at the single-cell level, thereby providing a more comprehensive understanding of cellular functions and characteristics [66, 68]. The development of CITE-seq marked a significant advancement in the field of single-cell research, providing a more comprehensive understanding of cell function and changes in both healthy and diseased states. Table 1 systematically compares several single-cell sequencing methods to illustrate the advancements in single-cell sequencing technologies.

**Table 1:** Comparison of Single-Cell Sequencing Methods

Year	Sequencing Method	Amplification Method	Cell Throughput	Capture Efficiency	Detected Gene Number	Advantages	Disadvantages	References
2011	STRT—seq	Reverse transcription and PCR amplification	Moderate	Moderate	Moderate	Can distinguish original transcripts from amplification duplicates by UMIs	Some details of library preparation complexity not elaborated	[69–71]
2012	CEL—seq2	In vitro transcription amplification after reverse transcription	Moderate to high	Moderate	Moderate	Reduces amplification noise with UMIs	Higher cost	[65, 71, 72]
2012	Smart—seq	Reverse transcription and PCR amplification	Moderate	Moderate	Moderate	Provides full—length transcript information	Prone to amplification bias and relatively lower throughput	[62, 73]
2013	Smart—seq2	Reverse transcription and PCR amplification	Moderate	High	High	Higher sensitivity and better coverage	Still lacks UMI for distinguishing amplification duplicates	[62, 64]
2014	MARS—seq	In vitro transcription amplification	High	Moderate	Moderate	Enables high—throughput analysis	Similar to CEL—seq2, in vitro transcription adds cost and complexity	[74, 75]
2015	Drop—seq	Reverse transcription and library construction in droplets	High	Moderate	Moderate	Cost—effective for large—scale single—cell analysis	Simultaneous inclusion of ERCC spike—ins is expensive	[66, 70, 74]
2015	10 × Genomics	Based on microfluidic technology and specific amplification chemistry	High	Varies by kit and experimental design	Varies by kit and experimental design	High throughput and wide application	Library preparation and sequencing costs may be relatively high	[76, 77]
2017	CITE—seq	Similar to traditional single—cell RNA—seq amplification methods with additional protein detection steps	Moderate	Varies for RNA and protein detection	Varies by experimental design	Provides multi—modal information	Protein detection may have limitations in sensitivity and specificity	[68, 78, 79]
2017	Cell Hashing	Depends on the scRNA-seq method used (e.g., 10 × Genomics)	Multiplexing Sample Barcoding	Very High (by pooling samples)	Same as underlying method	Dramatically reduces cost per sample; enables direct comparison across samples by pooling; removes batch effects	Requires additional antibody staining and optimization; barcode staining efficiency may vary	[80]
2019	SNARE-seq	PCR (for chromatin) & IVT (for RNA)	High	Moderate for RNA, High for ATAC	Moderate	First method to jointly profile chromatin accessibility and gene expression in the same single nucleus at low cost	Lower RNA detection sensitivity compared to dedicated scRNA-seq methods; complex data integration	[81]
2020	Paired-seq	Linear amplification	Very High	High	High	Ultra-low cost per cell; high data quality comparable to high-end methods	Requires specialized microfluidic equipment	[82]

Spatial transcriptomics technologies integrate gene expression with spatial location information, further compensating for the loss of spatial context inherent in single-cell technologies [83]. Single-cell sequencing has evolved from first-generation methods based on microplates to the current stage, which emphasizes high-throughput, multi-omics integration, and spatial resolution, becoming a core tool for deciphering cellular heterogeneity.

### Intersection between organoids and single-cell sequencing

Single-cell sequencing enables the systematic and quantitative assessment of how well the cell type composition in an organoid matches that of the corresponding human tissue through transcriptomic profiling [6, 84, 85]. For instance, in brain organoids, single-cell sequencing can identify whether key cell subtypes, such as excitatory neurons, inhibitory neurons, and astrocytes, are present and reveal their correspondence to specific stages of fetal brain development. Additionally, pseudo-temporal analysis can reconstruct the cell differentiation trajectories within organoids, determining whether they accurately recapitulate the lineage development pathways observed in vivo [86]*.* Although single-cell sequencing does not directly provide spatial information, its integration with spatial transcriptomic data enables the indirect inference of spatial organization patterns of different cell populations within organoids, thereby comprehensively validating their structural and functional biomimicry [87].

The maturity of organoids is often limited by the imperfections of the culture systems [88]. Single-cell sequencing can precisely identify abnormal cell states caused by the absence of or imbalance in signaling factors. Comparing the cellular atlases of organoids under various culture conditions can screen for key factors that promote the differentiation or functional maturation of specific cell subtypes [89]. For example, in liver organoids, single-cell sequencing has revealed that the addition of specific morphogenetic factors induces the expression of functional hepatic enzyme systems, significantly enhancing their metabolic maturity [90]. This data-driven optimization strategy, combined with technologies such as CRISPR base editing, enables the construction of disease models harboring specific mutations within weeks, significantly shortening research cycles [91]. Utilizing patient-derived iPSCs to circumvent species differences demonstrates unique value in both ethical and economic aspects [92].

Significant heterogeneity exists among cells, even within the same batch of organoids [93]. The high resolution of single-cell sequencing enables the identification of rare cell populations that are obscured in bulk analyses, such as endocrine precursor cells or tissue-specific stem cells [94, 95]. Single-cell sequencing can also detect unintended differentiated cells resulting from suboptimal culture conditions, providing direct evidence for optimizing culture protocols and enhancing the fidelity of organoids [96]. Compared to traditional 2D cell models, which fail to recapitulate the complex interactions and structures in vivo*,* and animal models, which are associated with high costs, long research cycles, and stringent ethical constraints, organoids—with their 3D structure—can faithfully recapitulate the organ microenvironment. When combined with single-cell sequencing, organoids can support precise analysis of cellular heterogeneity, thereby significantly enhancing experimental reproducibility and data reliability [97, 98].

The comprehensive integration of organoids and single-cell sequencing has driven fundamental research into the mechanisms underlying development and disease. It also has broad application prospects in drug development, as well as precision and regenerative medicine. With the continuous development and innovative integration of these two technologies, it is anticipated that they will bring further groundbreaking advancements to life science research.

## Demystifying organ development using organoids and single-cell sequencing

Advances in single-cell sequencing have facilitated dynamic research in organ development [99–102]. Organoids generated from iPSCs can simulate organ development [103]. The integration of single-cell sequencing with organoids has resolved issues in organ development [104]. Utilizing single-cell sequencing allows for the analysis of the similarity between organoids and real organs at the cellular, genetic, and functional levels, facilitating comparisons of heterogeneity among organs, understanding of the developmental differentiation of an organism through dynamic transcriptome profiling, and elucidation of interactions among organoid cells and with the surrounding matrix [105, 106].

### Cortical

The human brain is the most complex organ known among living organisms and represents the most advanced central nervous system (CNS) among life on earth. Previous studies on brain development and biology have often relied on rodent models, particularly mice and rats [107, 108]. While they have provided fundamental insights, the application of animal models in biomedical research is constrained by species differences. Additionally, 2D cell lines cannot recapitulate the hierarchical structure, spatial dimensions, cellular diversity, and intercellular interactions present in brain tissue. Moreover, the human brain exhibits more complex features, such as surface folding, forebrain expansion, layered organization, prolonged developmental processes, and richer cellular diversity, making it challenging to systematically elucidate human-specific gene regulatory mechanisms and pathways that control brain development [109–111]. These issues limit research on the human CNS. Furthermore, while postmortem brain tissue is considered valuable for studying the human brain, it only reflects the terminal state of the brain and cannot be extended to the early stages of brain development or disease, restricting the investigation of dynamic changes at the cellular level [27].

Recently, brain organoids have progressively overcome limitations in model construction, gradually expanding into the mechanistic investigation of neural development and neurodegenerative diseases [1]. Single-cell sequencing has played a crucial role in this process, with research revealing the ability of organoids to recapitulate the diversity of cell types, lineage differentiation trajectories, and gene expression characteristics during human brain development at high resolution [112]. Furthermore, one study conducted single-cell sequencing at multiple levels, including transcriptomics, epigenomics, and spatial transcriptomics, using cortical organoids [105], revealing that the diversity of cell types during cortical organoid development approximates that during endogenous embryonic development, thereby reaffirming the significant research value of brain organoids as in *vitro* developmental models in studying human cortical development [105, 113].

**Fig. 3:**
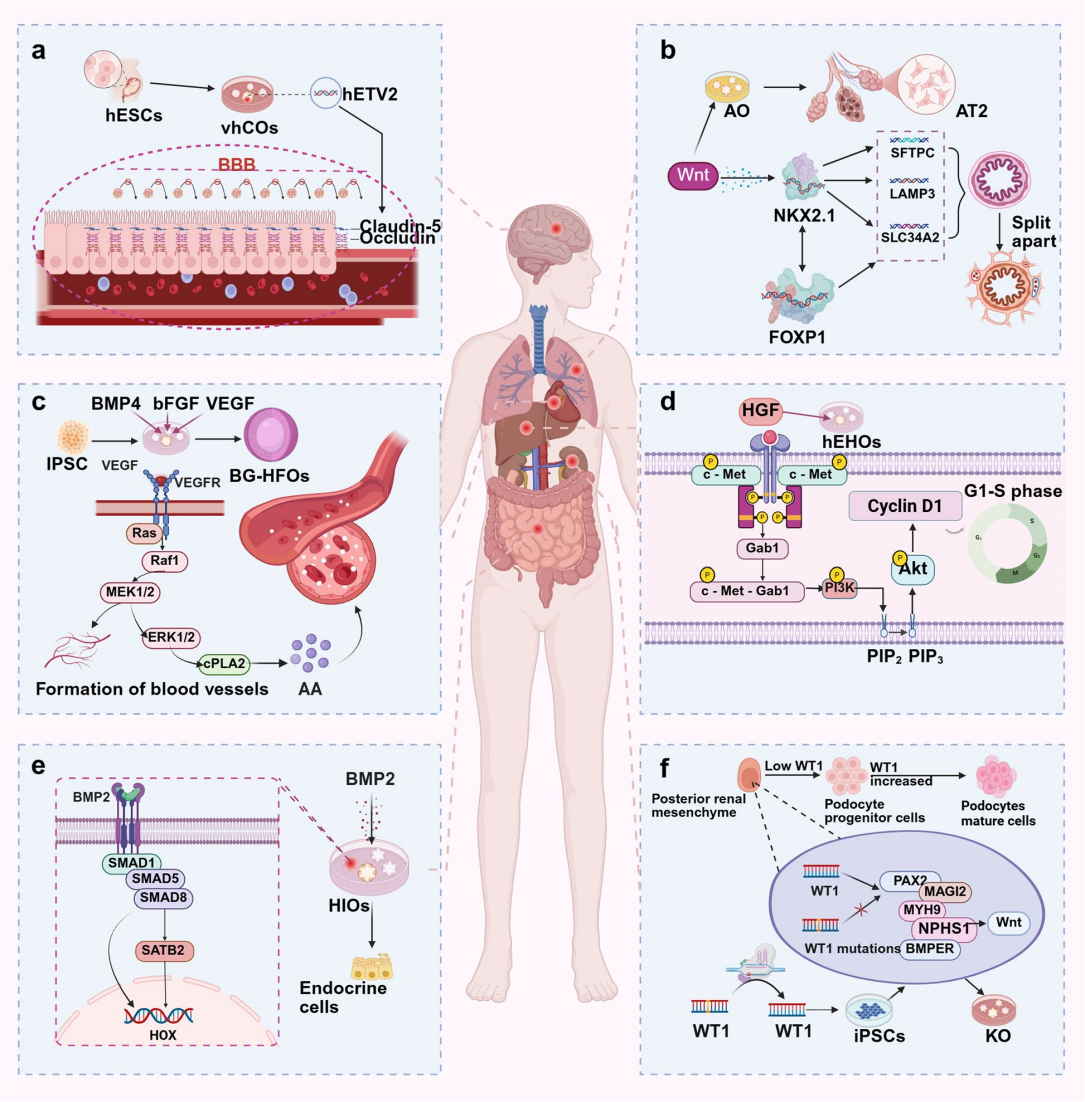
Functions and Regulatory Mechanisms of Signaling Pathways in Organoid Models. **a** The BBB regulates molecular permeability. Brain organoids were developed using hESCs expressing hETV2, which enhances BBB function by transcriptionally regulating the tight junction proteins CLDN5 and OCLN, reducing intercellular gaps, and preventing harmful substance entry. **b** In alveolar organoids(AO), Wnt signaling upregulates NKX2-1 expression, initiating alveolar gene expression programs. NKX2-1 directly binds to the promoters of SFTPC, LAMP3, and SLC34A2, promoting their transcription and regulating alveolar differentiation and maturation. Additionally, NKX2-1 cooperates with FOXP1 to construct a regulatory network coordinating alveolar development. **c** In Blood-generating HFO(BG-HFO) models, vascular development is facilitated by supplementing angiogenic factors, forming three-layered structures with a core, myocardial layer, and outer layer. Vascularization relies on VEGF and angiopoietin/TIE2 signaling, enhancing vascular permeability and establishing a stable blood circulation network to support CO functionality. **d** HGF activates signaling via its receptor c-Met. In liver organoids, exogenous HGF activates the PI3K/AKT pathway, promoting cyclin expression, driving cells from the G1 to the S phase of the cell cycle, and enhancing proliferation. **e** BMP signaling regulates gene expression via pSMAD1/5/8 activation. BMP2-treated intestinal organoids exhibit distal intestinal marker expression and generate colon-specific endocrine cells, highlighting the critical role of BMP in hindgut patterning. **f** WT1 is crucial for podocyte development and function during kidney organoid formation. WT1 expression increases with mesenchymal and podocyte progenitor development, localizing to mature podocytes. WT1 missense mutations impair the activation of target genes (MAGI2, MYH9, and NPHS1), delaying podocyte maturation and causing structural damage. Gene editing to correct WT1 mutations rescues podocyte phenotypes, illustrating WT1's pathogenic role in podocyte abnormalities

Regarding model construction, in addition to traditional iPSC-based organoids, a method for culturing human brain organoids directly from fetal tissue has been proposed, providing a new modeling strategy for organoid research and further enriching the technical pathways in this field [114]. However, existing brain organoids still face the challenge of lacking a functional vascular system, which limits their long-term culture and application in disease modeling. To overcome this limitation, extensive research has focused on achieving vascularization in brain organoids [115]. One study developed a technique to create human brain organoids with a functional vasculature-like system by expressing ETS variant transcription factor 2 (*ETV2*) in human ESCs (hESCs), which can regulate the promoter regions of genes encoding tight junction proteins, including claudin 5 (*CLDN5*) and occludin (*OCLN*), activating or inhibiting their transcription [116]. Upregulating the expression of *ETV2* may promote the expression of *CLDN5*, thereby enhancing intercellular tight junctions, reducing intercellular gaps, and preventing harmful substances from passing into the brain (Fig. 3a). This approach constructs organoids with a vascular-like system and enhances the expression of proteins related to the blood–brain barrier.

The co-culture strategy and organ fusion technology have also successfully generated brain organoids with complex vascular networks, providing a new platform for studying vascular–neural interactions [117]. For example, a novel protocol has been introduced for producing vascularized brain organoids by co-culturing human multipotent progenitor cells with human umbilical vein ECs (HUVECs), demonstrating for the first time that the vascular system in vascularized brain organoids can connect with host blood vessels to construct a new functional vascularized system [118]. Other studies have independently induced blood vessels and brain organoids, then fused them to generate vascularized brain organoids [119]. Brain organoids with unique cortical regions and anteroposterior patterns have also been generated, enabling more detailed investigations into human-specific brain development and diseases [120]. These organoids possess a complex vascular network that integrates well with brain cells and exhibit more neural cell precursors than unfused brain organoids, thereby overcoming the limitations of existing models and providing a new platform for studying vascular–neural interactions in brain development and function.

Single-cell sequencing has expanded the applications of brain organoids in disease research. In neurodegenerative disease research, integrating organoids with single-cell multi-omics analysis can dynamically capture disease-related changes in cellular state, abnormal protein aggregation, and microenvironmental perturbations, providing a highly biomimetic platform for mechanistic research and drug screening in conditions such as Alzheimer’s disease and Parkinson’s disease [121, 122].

Given the above, the comprehensive integration of brain organoids and single-cell sequencing has advanced our understanding of fundamental biological questions in human brain development, including neurogenesis and cortical regionalization. It has also significantly expanded the application of brain organoids in modeling neurodevelopmental disorders and neurodegenerative diseases. This technological combination is driving neuroscience research from static descriptions toward dynamic mechanism analysis, establishing a novel platform for uncovering the cellular and molecular foundations of human brain development and disease.

### Pulmonary

As essential respiratory organs of the human body, the lungs have complex structures and functions that have long been the focus of biological and medical research. Traditional in vivo and in vitro models exhibit significant limitations in recapitulating human lung tissues and present notable biological discrepancies. The emergence of lung organoids has dramatically advanced our understanding of the lung tissue microenvironment and its role in repair processes following lung injury [123].

Studies have used organoid models of human fetal alveolar development to reveal cell fate patterns and the mechanisms underlying neonatal respiratory diseases [124]. In recent years, significant progress has been made in the field of lung organoid research, particularly in the long-term culture of alveolar cells. The alveolar epithelium is primarily composed of alveolar type 1 (AT1) and type 2 (AT2) cells, which are responsible for gas exchange and surfactant secretion, respectively [125]. One study developed a novel culture system that achieved the long-term expansion of human AT2 cells and their differentiation into AT1 cells [126]. This breakthrough provides a reliable in vitro model for studying alveolar-related diseases, including idiopathic pulmonary fibrosis (IPF) and chronic obstructive pulmonary disease (COPD) [127].

The introduction of the air–liquid interface (ALI) culture approach has significantly enhanced the physiological relevance of lung organoids. By exposing organoids to an air environment, this approach more accurately simulates the in vivo airway microenvironment, thereby promoting key physiological processes, including the establishment of epithelial cell polarity, ciliogenesis, and mucus secretion [128]. ALI-cultured lung organoids have been successfully utilized in studying pathogen–host interactions, epithelial responses to air pollutants, and respiratory barrier functions, providing an essential tool for research on pulmonary epithelial biology [129, 130].

In mechanistic research on lung development, combining lung organoids with single-cell sequencing has revealed key regulatory networks [131]. Research indicates that Wnt signaling is a crucial upstream regulator of NK2 homeobox 1 (NKX2-1) levels [132, 133]. NKX2-1 binds directly to promoter regions of genes associated with alveolar differentiation-—surfactant protein C (*SFTPC*), lysosomal-associated membrane protein 3 (*LAMP3*), and solute carrier family 34 member 2 (*SLC34A2*)—promoting their transcription and thereby regulating the differentiation and functional maturation of alveolar cells [134]. Additionally, NKX2-1 interacts with other transcription factors, including forkhead box P1 (FOXP1), to coordinate the gene expression regulatory network during alveolar differentiation and maturation [135, 136]. Single-cell sequencing has been crucial in lung organoid research, providing a comprehensive analysis of the cellular composition and gene expression profiles of newly developed organoids (Fig. 3b). One study identified AT2 cells in differentiated alveolar organoids, further demonstrating that alveolar organoids can be used to study the differentiation and maturation processes of human alveolar cells [137].

Innovations in technical methods have also facilitated the development of lung organoids. A strategy has been established to direct the differentiation of human pluripotent stem cells (hPSCs) into distal lung organoids by sequentially differentiating them into definitive endoderm (DE), anterior foregut endoderm (AFE), ventral foregut endoderm, and lung bud organoids, ultimately forming lung organoids that recapitulate the process of lung development [138]. Another study established a stable culture system for human airway epithelial organoids, achieving the construction of a bipotent organoid model [123]. A further study established a stable human airway epithelial organoid culture system, achieving the construction of a dual-potent organoid system. Additionally, lung organoids constructed from hPSCs exhibited characteristics similar to those of embryonic developmental stages, including a multi-lineage cellular composition and complex tissue architecture. These organoids comprise multiple cell lineages, including mesenchymal and epithelial cells, and exhibit an ordered organization that resembles the structures of both proximal and distal airways [139].

Single-cell sequencing plays an indispensable role in lung organoid research. Applying single-cell sequencing to alveolar organoids enables the systematic elucidation of their cellular composition, lineage differentiation trajectories, and gene expression dynamics, effectively validating the model’s similarity to actual tissue [132]. One study utilized single-cell sequencing to identify AT2 cells within alveolar organoids and elucidate their differentiation pathways, providing high-resolution data to support research into lung development and regeneration mechanisms [140]. As the technology continues to mature, lung organoids have expanded from fundamental developmental biology research into applied fields, including disease modeling and drug screening [141]. These advances provide a robust platform for deepening understanding of lung regeneration mechanisms, developing targeted therapeutic strategies, and advancing personalized medicine, heralding broad prospects in respiratory disease research and treatment development.

### Cardiac

The human heart is a functional organ that develops during embryogenesis [142]. Reproducing the early stages of heart development in vitro presents particular challenges. Cardiac organoids, serving as crucial in vitro models for studying heart development and diseases, are currently primarily constructed from hiPSCs, offering new insights into heart development. Cardiac organoids began to emerge more frequently after 2017 [143, 144]. They were initially constructed using cardiomyocytes (CMs) derived from hiPSCs in conjunction with primary stromal cells, HUVECs, and human cardiac fibroblasts (CFs) [145, 146]. With advances in and the application of iPSCs, most contemporary cardiac organoids are formed either by directly differentiating embryoid bodies derived from iPSCs into CMs or by co-culturing multiple cardiac cell types derived from iPSCs [147–150].

Physiologically, human cardiac organoids essentially mimic the in vivo distribution of cells, responses to external stimuli, and electrophysiological functions [151, 152]. However, primary stromal cells do not represent the genetic characteristics of specific patients, which limits the accuracy of disease modeling. To address this limitation, cardiac organoids have been constructed using CMs, CFs, and ECs derived from hiPSCs [153, 154]. This approach replicates patient-specific genetic variations and allows for the introduction of controlled genetic mutations or variations through gene-editing techniques, providing a means to model disease states and cardiac functions [155]. Cardiac organoids offer a foundation for studying cardiac development by simulating embryonic tissue and stage-specific 3D self-organizing structures in vitro.

One study established blood-generating heart-forming organoids by precisely supplementing cell cultures with multiple factors that promote hematopoiesis and vascular development: bone morphogenetic protein 4 (BMP4), basic fibroblast growth factor (bFGF), and vascular endothelial growth factor (VEGF). This approach ensures the orderly formation of organoids, successfully creating a three-layered structure comprising a core, a myocardial layer, and an outer layer. Each layer has distinct functions and collaborates to simulate the processes of heart and blood development, effectively recreating the embryonic anatomical layout [156]. In blood-generating heart-forming organoids, the angiogenesis process involves the VEGF signaling pathway and the classical TEK receptor tyrosine kinase (TEK/TIE2) signaling pathway. Specifically, VEGF binds to its receptor kinase insert domain receptor (KDR/VEGFR), which then activates the upstream Ras protein and further triggers the activation of the Raf-1 proto-oncogene, serine/threonine kinase (RAF1), initiating a cascade of signaling events. RAF1 activates downstream mitogen-activated protein kinase kinases 1 (MAP2K21/MEK1) and 2 (MAP2K2/MEK2), which then activate mitogen-activated protein kinases 1 (MAPK1/ERK2) and 3 (MAPK3/ERK1). This signaling pathway promotes EC migration and the formation of newly sprouting capillaries [157, 158]. ERK1 and ERK2 also activate cytosolic phospholipase A_2_, leading to the release of arachidonic acid (Fig. 3c), which is then metabolized into prostaglandins and other molecules, thereby enhancing vascular permeability and supporting the dynamic regulation of vascular networks. This signaling pathway facilitates the establishment of a stable circulatory network within organoids, ensuring cellular metabolism and function, and thereby improving the physiological simulation of cardiac organoids [159]. Single-cell sequencing has been used to examine the gene expression profiles of blood-generating heart-forming organoids, accurately identifying myocardial cells, various EC types, hematopoietic progenitor cells, erythrocyte precursors, and leukocyte precursors [156].

Additionally, one study reported the successful cultivation of “miniature hearts” from stem cells that resembled early human embryonic hearts [160]. Measuring just 0.5 mm in diameter, these “miniature hearts” are organoids containing both myocardial and epicardial cells and can contract like human heart chambers when electrically stimulated. Another study developed multi-chamber heart organoids, which included the right ventricle (RV), left ventricle (LV), atrium, outflow tract (OFT), and atrioventricular canal (AVC), ultimately generating progenitor cell subpopulations with anterior and posterior heart chambers [161]. Single-cell sequencing and functional characterization revealed that the gene expression profiles of these heart organoids corresponded to different chambers during in vivo development, providing a platform for studying the coordinated development of organs.

The hPSC-derived heart-forming organoids encompass various aspects of the concurrent development of the heart, vascular system, and foregut, achieving the co-development of cardiac, endothelial, and multipotent hematopoietic tissues analogous to the in vivo embryonic hematopoietic region, thereby advancing in vitro hematopoiesis research [162]. Previously, the lack of humanized cardiac models that resemble myocardial injury and inflammatory responses has limited research into therapeutic drugs for myocardial ischemia/reperfusion (I/R) injury. One study also developed ventricular heart organoids from hiPSCs to simulate I/R injury induced by hypoxia/reoxygenation (H/R) [163]. The further development of these heart organoid models will facilitate a deeper exploration of the cooperative mechanisms of cardiac development and hematopoiesis, providing cellular and molecular targets for cardiovascular disease pathology.

Combining single-cell sequencing and cardiac organoids has deepened our understanding of cardiac development and provided new tools and perspectives for studying developmental disorders. As these techniques continue to advance, cardiac organoids will have an increasingly critical role in cardiovascular disease mechanism research, drug screening, and personalized medicine.

### Liver

The liver is a vital organ responsible for producing and storing amino acids and vitamins, as well as performing various functions, including metabolism and detoxification. It comprises two types of epithelial cells: hepatocytes and bile duct cells. Monocultured hepatocytes do not remain stable in the long term. Multicellular liver tissue cultures are better suited to drug screening, disease modeling, and transplantation therapy than hepatocyte monocultures. However, these model systems are non-proliferative and have limitations in scalability [164]. In 2013, one study successfully established a mouse-derived liver organoid system by adding components such as hepatocyte growth factor (HGF), epidermal growth factor (EGF), and fibroblast growth factor (FGF) to the culture system [165]. However, significant differences exist between the effects of various drugs in animal models and in clinical use [166]. Human liver organoids derived from adult or fetal hepatocytes, hepatocyte-like cells (HLCs) derived from hiPSCs, and reprogrammed human hepatocytes can reproduce the key morphological, functional, and gene expression characteristics of mature hepatocytes in vivo [167].

Notably, some studies have established a stable culture system for adult hepatocyte organoids by optimizing culture conditions, thereby addressing the limitation in maintaining the functions of primary hepatocytes over the long term. In 2019, one study reported a newly developed method that utilizes a fully defined serum-free and feeder-free medium to produce hESC-derived expandable hepatic organoids [168]. These organoids stably maintained the bipotent phenotype of hepatic stem/progenitor cells, allowing them to differentiate into functional hepatocytes or cholangiocytes. Another study combined microengineering techniques with stem cell biology to generate well-functioning liver organoids from hiPSCs [169]. In 2022, a study thoroughly explored and optimized the culture medium for hiPSC-derived liver organoids, identifying four essential growth factors needed for the various stages of liver organoid differentiation: activin A, BMP4, oncostatin M (OSM), and HGF [170].

Liver organoids successfully recapitulated the zonation phenomenon of liver lobules, a critical feature for hepatic metabolic functions [171]. Single-cell sequencing has revealed the central regulatory role of the Wnt/catenin beta 1 (CTNNB1/β-catenin) signaling gradient in establishing zonation [172]. Analyzing the spatial distribution of gene expression in hepatocytes within organoids revealed that subpopulations of hepatocytes with distinct metabolic functions exhibit distribution patterns similar to those in vivo, providing new insights into the molecular basis of liver zonation [173]. The liver organoids demonstrate significant potential in high-throughput functional genomics studies. One study utilized liver organoids to conduct large-scale CRISPR screens, systematically identifying key gene networks involved in liver development and disease [174]. These studies have deepened our understanding of liver biology and also provided valuable resources for drug target discovery.

HGF primarily initiates signal transduction by binding to its transmembrane receptor, MET proto-oncogene, receptor tyrosine kinase (MET/c-Met) [175]. Upon binding to HGF, c-Met dimerizes, activating its intracellular tyrosine kinase activity. In a standard hepatic organoid culture system, the addition of exogenous HGF results in a rapid increase in the phosphorylation of c-Met, indicating the activation of its signaling pathway. This phosphorylation recruits a series of downstream signaling molecules, including GRB2-associated-binding protein 1 (GAB1). Additionally, the activated c-Met/GAB1 complex can activate the phosphoinositide 3-kinase (PI3K)/protein kinase B (AKT) signaling pathway [176]. PI3K phosphorylates phosphatidylinositol-4,5-bisphosphate (PIP2) on the cell membrane to produce phosphatidylinositol-3,4,5-trisphosphate (PIP3), a secondary messenger that recruits AKT to the cell membrane, where it is phosphorylated (activated). AKT activation promotes the expression of proteins, such as cyclin D1 (*CCND1*), facilitating the progression of hepatic organoid cells from the G1 to the S phase of the cell cycle, thereby promoting their proliferation [177]. Cell proliferation is crucial during the development of hepatic organoids, allowing them to increase in size and form structures resembling liver tissue (Fig. 3d).

One study also employed co-culture techniques to establish a culture model of adult mouse hepatobiliary organoids, significantly enhancing their long-term expansion capability [178]. Another study reported tracking temporal transcriptomic and phenotypic changes in cultures of human-derived adult hepatocytes, comparing them to fetal-derived hepatocytes, which led to the first observation of the lack of proliferation initiation and inhibition of lipid metabolism in adult hepatocyte-based organoid cultures [90]. A further study observed that supplementation with growth-related factors, such as interleukin 6 (IL6) and nuclear receptor subfamily 1 group H member 4 (NR1H4/FXR), enabled the in vitro culturing of organoids with adult hepatocyte characteristics. Additionally, single-cell sequencing of the fetal and adult liver highlighted their high complexity and cellular heterogeneity, identifying new cell subpopulations. An in-depth analysis of individual cells precisely identified regulatory genes crucial for this developmental maturation process, providing critical evidence for further research into the standard development mechanisms of the liver and the pathogenesis and therapeutic strategies for related liver diseases.

### Intestinal

Intestinal development is a highly complex and fascinating biological process, with intestinal organoids playing a crucial role in this field. Advanced single-cell sequencing technologies have been used to investigate the differentiation trajectories of intestinal epithelial cells in detail. In 2009, one study reported the successful in vitro cultivation of mouse leucine-rich-repeat-containing G protein-coupled receptor 5 (LGR5)^+^ intestinal stem cells into mouse small intestinal organoids, recapitulating the 3D structures of the crypt- and villus-like epithelial regions [23]. The subsequent optimization of the culture conditions enabled the accurate simulation of the physiological state of mouse small intestinal epithelium, allowing for long-term in vitro culture [179]. Additionally, one study demonstrated the in vitro simulation of embryonic intestinal development using hPSCs, where hPSC-derived DE differentiated into midgut and hindgut endoderm, thereby facilitating gut tube-like morphogenesis [180].

The in vitro cultivation of isolated intestinal stem cells into intestinal organoids depends on recreating the microenvironment needed for the proliferation, differentiation, and migration of intestinal stem cells. In the native intestine, the niche for intestinal stem cells is a complex structure composed of various intestinal cells, primarily Paneth cells, intestinal stromal cells, fibroblasts, lymphocytes, and nerve cells [181]. One study utilized single-cell transcriptomic mapping of respiratory and gastrointestinal (GI) endodermal organogenesis to elucidate cellular states and benchmark human intestinal organoids, demonstrating the influence of mesenchymal stem cells (MSCs) on intestinal development [182]. Another study demonstrated that activation of the bone morphogenetic protein (BMP) signaling pathway generates colonic organoids with early hematopoietic features [183]. With their capacity for self-renewal and multidirectional differentiation, hPSCs can be used to generate a diverse cell types both in vitro and in vivo. However, existing differentiation protocols often suffer from low efficiency and functional inadequacy. To overcome these challenges, the roles of different signaling pathways, particularly the BMP signaling pathway, in stem cell differentiation have been explored. The BMP signaling pathway was found to be rapidly activatable, effectively guiding hPSCs toward differentiation into intestinal organoids.

BMPs belonging to the transforming growth factor-beta (TGF-β) superfamily play a crucial role in human intestinal development [184]. Research on intestinal organoids has demonstrated that bone morphogenetic protein 2 (BMP2) significantly increases the levels of phosphorylated SMAD family members 1 (p-SMAD1), 5 (p-SMAD5), and 8 (p-SMAD8), a key step in the BMP signaling pathway. BMP2 induces the expression of SATB homeobox 2 (*SATB2*), a chromatin remodeling protein that influences gene expression patterns. BMP2 also promotes the expression of posterior homeobox (HOX) genes, which are responsible for establishing cell fate along the anterior–posterior axis and play significant roles in organ morphogenesis during embryonic development. Therefore, BMP2 affects intestinal organ morphogenesis by regulating the expression of these factors, thereby playing a crucial role in the patterning of the intestinal tube. Organoids treated with BMP2 exhibit marker expression patterns consistent with the distal intestine after long-term culture in vitro [185]. When transplanted in vivo, human intestinal organoids and colon organoids retain their regional characteristics, expressing region-specific markers and hormones, with gene expression patterns that are highly consistent with those of the corresponding human intestinal regions. Furthermore, colonic organoids treated with BMP2 can produce colon-specific endocrine cells, further elucidating the critical role of the BMP signaling pathway (Fig. 3e).

The intestinal epithelium harbors various rare cell types that, despite their extremely low proportions, play crucial functional roles, which are difficult to effectively replicate with traditional models [186]. The integration of intestinal organoids and single-cell sequencing technologies has provided a breakthrough tool for the study of these cells. Among them, the research on enteroendocrine cells (EECs) is most representative [187]. These cells constitute approximately 1% of the intestinal epithelial cells but are key components of the gut-brain axis, regulating various physiological functions including appetite and insulin release [188]. Single-cell sequencing technology, through systematic analysis of human intestinal development, has revealed the complexity of signaling pathways like EGF/ERBB and identified key factors involved in the differentiation of rare cell types [188, 189]. The construction of the single-cell transcriptomic atlas of human EEC tissues has significantly advanced the in-depth study of rare intestinal cells, providing a crucial resource for understanding the functions of these cells under both physiological and pathological conditions [190].

The development of genome editing technologies, such as CRISPR/Cas9, has transformed intestinal organoids from developmental models into functional research platforms [191, 192]. Studies have successfully simulated hereditary intestinal diseases, such as cystic fibrosis and familial adenomatous polyposis, by introducing specific gene knockouts or pathogenic mutations in organoids [191, 193]. These disease-specific organoids exhibit highly humanized characteristics, providing reliable tools for disease mechanism research and drug screening, effectively compensating for the significant physiological differences between traditional animal models and humans. Particularly noteworthy is the use of CRISPR technology to genetically label specific cell types, LGR5 + intestinal stem cells and EECs, enabling researchers to track the fate determination and differentiation pathways of these cells in real-time during organoid development [23, 194]. This technology revealed the dynamic changes in the lineage of intestinal cells. It enabled the visualization and sorting of rare cells through the construction of a gene reporter system, providing high-purity cell samples for subsequent functional studies [195]. The combination of intestinal organoids and single-cell technologies has significantly deepened our understanding of intestinal development and the mechanisms that maintain homeostasis. It has also led to breakthroughs in precision medicine and the development of novel drugs for intestinal diseases.

### Renal

The mammalian kidney comprises thousands to millions of nephrons and a branching network of collecting ducts connected to them [196, 197]. During embryonic kidney development, nephron and ureteric bud (UB) progenitors mutually induce further differentiation of progenitor cells and branching of the collecting duct, ultimately forming a complex nephron-collecting duct network structure [198, 199]. Kidney organoids are 3D cell aggregates derived from in vitro stem cell culture, replicating key aspects of kidney development and forming glomerular structures [200]. Recently, studies on kidney organoids have evolved from primarily developing nephron organoids to modeling UB or collecting duct organoids, thereby simulating human kidneys [201–204]. Advances in kidney organoid technology have led to the formation of organoids with more specialized cell types, reduced heterogeneity, and increased structural complexity [205]. One critical factor in this process is the interaction between the UB and the metanephric mesenchyme (MM). The UB originates from the Wolffian duct, while the MM forms as the UB invades the embryonic body [185]. The interaction between the UB and the MM promotes kidney morphogenesis and determines the final structure of the nephron.

Kidney organoids have evolved from early models primarily establishing nephron organoids to more complex organoids capable of simulating ureteric buds or collecting ducts [206]. These advances have enabled kidney organoids to incorporate more specialized cell types, exhibit increased structural complexity, and significantly reduce heterogeneity. Among these developments, simulating interactions between the UB and MM has become critical, as this process plays a central role in kidney morphogenesis and the formation of nephron structure in vivo [207]. However, early kidney organoids exhibited notable limitations, including the absence of a vascular system and ineffective connections between nephrons and collecting ducts. To overcome these challenges, researchers have developed multiple innovative strategies. Flow culture on microfluidic chips significantly enhances the maturity of kidney organoids, as evidenced by the development of more mature capsules and tubular compartments, along with improved cell polarity and the expression of adult genes [208, 209]. Furthermore, kidney organoids cultured using kidney extracellular matrix hydrogels exhibit extensive vascular networks and self-derived ECs [210]. Single-cell sequencing analysis confirms their more mature glomerular development patterns and higher similarity to human kidneys [211].

Single-cell sequencing technology has had a pivotal impact on kidney organoid research, particularly in deciphering key transcription factor networks. Taking *WT1* as an example, this transcription factor is crucial for podocyte development and functional maintenance [212]. *WT1* expression increases with the development of mesenchyme and podocyte progenitors, eventually becoming restricted to mature podocytes [213, 214]. One study utilized single-cell sequencing of fetal kidneys and kidney organoids to identify a range of WT1 target genes essential for podocyte development and structural maintenance, including BMP-binding endothelial regulator (*BMPER*), paired box 2 (*PAX2*), and membrane-associated guanylate kinase, WW and PDZ domain containing 2 (*MAGI2*), which regulate the Wnt signaling pathway, myosin heavy chain 9 (*MYH9*), which maintains actin filament organization; and NPHS1 adhesion molecule, nephrin (*NPHS1*), which regulates cell junction assembly [212]. Heterozygous missense mutations in *WT1* prevented it from activating target genes like *MAGI2*, *MYH9*, and *NPHS1*, leading to delayed podocyte development and cellular structural damage. This observation has been confirmed through single-cell sequencing and functional analysis of kidney organoids derived from iPSCs from patients with *WT1* mutations. Moreover, correcting the *WT1* mutation in patients using the CRISPR/Cas9 system could rescue the podocyte phenotype, underscoring the pathogenic role of *WT1* mutations in abnormal podocyte development [207]. Another study found that *WT1* mutations disrupting the podocyte phenotype were often limited to the role of single genes. A further study has elucidated the epigenomic landscape of *WT1* in human podocyte development at a single-cell resolution from a systemic perspective, revealing the key mechanistic role of WT1 in regulating podocyte development and maintaining podocyte structure and function during kidney development and kidney organoid differentiation [212]. These findings highlight the advantages and value of kidney organoids as models of the human kidney (Fig. 3f).

With the deep integration of single-cell multi-omics analysis and gene editing technologies, kidney organoids have broad application prospects in simulating kidney development, studying disease mechanisms, and drug screening [215]. Future research directions include improving the vascularization of organoids, enhancing structural complexity, and better simulating the physiological functions of the kidney [215]. The combination of kidney organoids and single-cell technology will continue to drive the transformation of kidney developmental biology from descriptive research to mechanistic understanding, providing breakthroughs for precision medicine in kidney diseases.

In summary, the deep integration of single-cell sequencing and organoid technology has opened up new avenues for understanding the cellular and molecular basis of organ development. Through systematic application in key organ models such as the brain, lung, heart, liver, intestine, and kidney, this technological combination has revealed cellular heterogeneity, lineage differentiation trajectories, and gene regulatory networks at high resolution, demonstrating its advantages in simulating human-specific developmental processes and complex disease mechanisms. Current models still face challenges in vascularization, functional maturity, and standardization, but engineered culture strategies and multi-omics integrated analysis are continuously driving them toward higher physiological relevance. In the future, with the further integration of cutting-edge technologies, for instance, spatial transcriptomics, gene editing, and live imaging, organoids will transition from static simulation to dynamic analysis, continuously providing a robust platform for developmental biology, disease mechanism research, and precision medicine, ultimately driving a paradigm shift in life science research.

## Single-cell sequencing deciphers the heterogeneity of cancer organoids

As a significant disease impacting human health, cancer has become the second leading cause of death globally [216]. Tumors are complex amalgams of malignant, immune, and stromal cells. The tumor microenvironment encompasses both tumor-promoting and tumor-suppressing signals that regulate tumor growth and evolution. During tumor progression, cancer cells undergo multiple rounds of division and proliferation, resulting in a series of signal transductions, which influence cell growth, migration, and invasion [217]. These processes contribute to inter-tumor and intra-tumor heterogeneity [218], which leads to diversity in signaling pathways and drives phenotypic variation [219].

### Lung cancer

Lung cancer (LC) exhibits significant complexity and heterogeneity, encompassing non-small cell LC (NSCLC) and small cell LC, with diverse mutational profiles. Tumor heterogeneity poses a considerable challenge in LC treatment, as substantial genetic, epigenetic, and phenotypic differences exist among tumors, complicating the development of precise and effective treatment methods [174]. Organoid models retain the genetic, transcriptional, morphological, and drug sensitivity characteristics of the parental tumors [19]. The tumor microenvironment in LC organoids includes various cell types, including immune, vascular endothelial, and stromal cells. Single-cell sequencing can elucidate the roles of these cells in the development of tumors. For example, tumor-associated macrophages (TAMs) exhibit different polarization states within LC organoids, where M2-type TAMs promote tumor cell proliferation and metastasis by secreting cytokines, name a interleukin 10 (IL-10) [220].

The Wnt/β-catenin pathway plays a pivotal role in LC progression [221], with Wnt family member 3 A (Wnt3A) acting as a key activator. The binding of Wnt3A to frizzled class receptors and co-receptors LDL receptor-related protein 5/6 (LRP5/6) at the cell membrane initiates a cascade of complex and ordered signaling events [222]. This cascade involves the activation of disheveled segment polarity proteins, which disrupt the stability of the β-catenin destruction complex composed of AXIN, APC regulator of Wnt signaling pathway (APC), GSK3β, and casein kinase 1 (CK1) [223, 224]. Normally, GSK3β activity phosphorylates β-catenin, marking it for ubiquitin–proteasome degradation. However, Wnt3A signaling prevents its degradation, allowing β-catenin to accumulate in the cytoplasm. Then, β-catenin translocates to the nucleus, interacting with the T cell factor/lymphoid enhancer factor (TCF/LEF) family of transcription factors [225], forming a transcriptionally active complex that targets promoters of genes crucial for stemness and proliferation, cellular myelocytomatosis (*c-Myc*), *CCND1*, and *survivin* [221]. For example, as an important oncogene, c-Myc activation can drive cell cycle progression, promote metabolic reprogramming, and enhance cell proliferation capacity [226]. *CCND1* helps to regulate the transition from the G1 phase to the S phase of the cell cycle, accelerating cellular proliferation [227]. By activating the expression of these genes, Wnt3A successfully enhances the activity of the Wnt/β-catenin pathway [228], establishing a robust molecular foundation for maintaining stem cell pluripotency, which endows tumor cells with a strong potential for self-renewal and proliferation advantage, facilitating the progressive evolution of LC cells from normal to malignant states. By enhancing tumor cell proliferation, invasion, and metastatic potential, this process thereby exerts a profound influence on LC pathogenesis, clinical outcomes, and ultimately, the efficacy of therapeutic interventions.

Establishing a biobank of LC organoids facilitates the screening of anticancer drugs and uncovers potential differences in resistance [229], as well as varied responses to erlotinib and crizotinib [220, 228]. In immunotherapy, co-culture models of organoids and immune cells enable the identification of immune activation strategies and the study of tumor immune microenvironment responses. Moreover, LC organoids can facilitate personalizing cancer treatment by assessing drug toxicity through comparisons with normal lung organoids, providing patients with precise therapeutic methods.

### Breast cancer

Breast cancer (BC) is a highly heterogeneous disease with multiple molecular subtypes, including luminal A, luminal B, human epidermal growth factor receptor 2 (HER2)-positive, and triple-negative (TNBC). Traditional 2D cell cultures and animal models often fail to replicate BC complexity [230]. BC organoids effectively preserve the heterogeneity of tumor tissues, with distinct morphological, cellular, and gene expression profiles across different subtypes, reflecting intratumoral diversity [231]. They faithfully retain the genetic characteristics of tumors, including mutations, gene amplifications, and chromosomal abnormalities, which are crucial for studying BC pathogenesis, predicting therapeutic responses, and developing personalized treatments. Single-cell sequencing has revealed that BC organoids retain the characteristics of primary BCs (PBCs) to varying degrees across different molecular subtypes. While BC organoids can closely retain the characteristics of PBCs in hormone receptor (HR)-positive and HER2-positive BC, they differ significantly from PBCs in stemness, hypoxia-related pathways, and drug sensitivity in TNBC [232]. This discrepancy may be related to oxygen levels in the culture conditions. Single-cell sequencing can further delineate the characteristics of various cell subpopulations within the tumor microenvironment, among others immune cells and stromal cells.

In approximately 15%–20% of BC cases, overexpression or amplification of the *HER2* gene plays a crucial role in tumorigenesis. In BC organoids, the HER2 signaling pathway is typically aberrantly activated through heterodimerization with other HER family members, activating downstream pathways such as Ras/Raf/MEK/ERK and PI3K/AKT/mTOR, which promotes tumor cell proliferation, migration, and invasion [233, 234]. Despite the significant therapeutic efficacy of HER2-targeted drugs like trastuzumab and pertuzumab in HER2-positive BC, resistance remains a clinical challenge. Therefore, the regulation and resistance of the HER2 signaling pathway in BC organoids are vital for developing new anti-HER2 therapeutic strategies. The PI3K/AKT/mTOR pathway is frequently hyperactivated in BC [235, 236], which is closely associated with tumor cell proliferation, metabolism, and resistance [237]. In BC organoids, various factors can activate this pathway, overexpression of *HER2* or loss of phosphatase and tensin homolog (PTEN). Modulating the PI3K/AKT/mTOR pathway significantly impacts the growth and survival of BC organoids [238]. Multiple inhibitors targeting this pathway are currently under development and evaluation in clinical trials.

In BC, patient-derived organoids (PDOs) are considered promising models to overcome the limitations of cell lines and patient-derived xenografts (PDXs) [239]. One study aimed to validate the efficacy of PDOs in predicting patient drug responses by establishing a biobank of BC PDOs [240, 241]. Another study utilized PDOs to explore personalized treatments for refractory BC. It successfully demonstrated that the sensitivity of PDOs to microtubule-targeting drugs can predict distant recurrence-free survival in invasive breast cancer treated with adjuvant chemotherapy [242]. Its findings suggest that PDOs can inform personalized treatment decisions for patients with advanced BC.

### Liver cancer

Liver cancer, as a severe malignant tumor, holds a significant position in both incidence and mortality rates globally. The most common type of primary liver cancer is Hepatocellular Carcinoma (HCC), while cancer originating from the bile ducts is termed Cholangiocarcinoma [243]. HCC is a malignant disease that poses a serious threat to human health, with high incidence and mortality. A liver cancer organoid biobank comprised of 399 tumor organoids derived from 144 patient samples revealed a degree of mutation concordance between tumor tissues and organoids [244]. However, some exhibited tumor evolution during passaging, highlighting cellular heterogeneity. Organoids from different patients and regions of the same tumor showed differences in gene expression and mutation profiles, further underscoring this heterogeneity [245]. The complex tumor microenvironment in primary liver cancer can be further explored using organoid models to study intercellular interactions [246].

Multiple signaling pathways and associated proteins play critical roles during HCC development and progression. The overexpression of cellular jun-transforming protein (c-Jun) can mediate resistance to lenvatinib via the JUN N-terminal kinase (JNK) and β-catenin signaling pathways. The synthesized compound PKUF-01, composed of lenvatinib and veratramine, exhibited synergistic inhibitory effects on resistant cells. The histone deacetylase (HDAC) inhibitor suberoylanilide hydroxamic acid (SAHA) upregulates the expression of *PTEN* and inhibits the AKT signaling pathway, thereby reversing lenvatinib resistance in HCC cells [244]. In minimal residual disease (MRD) of HCC, programmed cell death 1 ligand 1 (PD-L1)-positive M2 macrophages interact with cancer stem cells through the TGF-β signaling pathway, sustaining tumor cell persistence. Combined blockade of PD-L1 and TGF-β signaling pathways effectively prevents HCC recurrence. The aberrant activation or dysregulation of these pathways and proteins collectively contributes to HHC development, progression, drug resistance, and recurrence.

Utilizing HCC organoid models, research has proposed that collaboration among different cellular subpopulations within tumors leads to drug resistance, offering new insights and strategies for treating HCC. Combining patient-derived tumor organoids with single-cell sequencing and drug screening assays has revealed drug resistance mechanisms in HCC [247]. Pharmacogenomic analysis of organoids has enabled the identification of biomarkers predictive of drug response, to name a few lenvatinib resistance. In-depth investigations of resistance can facilitate the development of novel combination therapies [248]. For example, combining lenvatinib with HDAC or AKT inhibitors synergistically inhibited tumor cell proliferation and induced apoptosis, enhancing therapeutic efficacy [249]. This approach supports personalized medicine in treating HCC by tailoring strategies to the unique characteristics of a patient’s tumor, including genetic mutations, gene expression profiles, and drug sensitivities, thereby improving treatment precision and effectiveness.

### Gastric cancer

Gastric Cancer (GC) ranks among the most prevalent and deadly malignant tumors worldwide. GC organoids serve as tumor surrogates, effectively mimicking the tumor microenvironment and facilitating research into tumor biology. They overcome the limitations of traditional GC cell lines, which often fail to represent the characteristics of the original tumor tissue accurately [250]. The application of high-throughput single-cell sequencing to tumor organoids has expedited the identification of tumor driver mutations, dysregulated programs, and molecular subtypes [251]. One study used a diffuse-type GC PDO-monocyte/macrophage co-culture system, discovering that aurora kinase inhibitors (AURKi) induce senescence in diffuse GC. Another study demonstrated that senescent cells secrete C–C motif chemokine ligand 2 (CCL2/MCP-1), which triggers the polarization of locally accumulated monocyte/macrophages towards the M2 phenotype, creating an immunosuppressive microenvironment that weakens the innate immune capacity of macrophages to eliminate tumor cells [252].

GC involves several critical signaling pathways. The MEK/ERK and signal transducer and activator of transcription 3 (STAT3) pathways play crucial roles in the growth and survival of gastric precancerous lesions. Dual inhibition of these pathways can suppress the stem cell and dysplastic features of precancerous lesions, offering new strategies for treating GC [253]. The claudin 18 (*CLDN18*)-Rho GTPase activating protein 26 (*ARHGAP26*) fusion gene is frequently recurrent in diffuse GC, inducing the formation of characteristic “signet-ring cells” and promoting the activation of ras homolog family member A (RHOA) and its downstream signaling, notably the focal adhesion kinase (FAK) and YAP signaling pathways. Co-inhibition of the FAK and YAP/TEAD pathways significantly impedes tumor growth, providing a basis for evaluating the therapeutic potential of related inhibitors [254]. Additionally, N-acetyltransferase 10 (*NAT10*) is overexpressed in GC and is associated with poor prognosis. NAT10 regulates RNA dynamics through condensate formation and interacts with splicing factor serine- and arginine-rich splicing factor 2 (SRSF2), affecting the splicing pattern of the m^6^A reader YTH N^6^-methyladenosine RNA binding protein F1 (YTHDF1) [255].

In drug screening, establishing GC organoid biobanks enables in vitro personalized drug testing [256]. This approach can help identify gene expression signatures associated with chemosensitivity or resistance, facilitating the development of predictive models. One study using GC organoids showed that those sensitive to 5-fluorouracil (5-FU) or oxaliplatin exhibited upregulation of tumor suppressor genes or pathways, whereas organoids resistant to 5-FU exhibited increased proliferation and invasion [256]. In immunotherapy, it has been observed that drug-induced senescent cells can affect macrophage function. For example, a study using diffuse GC PDO-monocyte/macrophage co-cultures demonstrated that AURKi-induced senescent cells secreted CCL2/MCP-1, promoting the M2 polarization of monocytes/macrophages and weakening their innate immune capacity, suggesting a new therapeutic strategy [252]. Another study examined 5-FU-resistant GC organoids and identified KH RNA binding domain-containing signal transduction associated 3 (KHDRBS3) as a promising biomarker for predicting treatment response and prognosis [257]. In personalized medicine, GC organoids reflect patient-specific tumor characteristics, aiding in the tailored selection of chemotherapeutic agents. Different GC subtypes respond variably to drugs. For example, patients with poorly differentiated intestinal-type GC may benefit from combined 5-FU and veliparib treatment [254]. Organoid models hold the potential for advancing precision treatment in GC.

### Colorectal cancer

Traditional research models face limitations in simulating the complexity of CRC, whereas the advent of organoid models has brought significant breakthroughs to CRC. CRC organoids encompass tumor types in various states [252]. Single-cell sequencing of CRC organoids has revealed the heterogeneity of tumor cells, demonstrating differences in gene expression and cellular functions among different PDOs. For example, single-cell sequencing of CRC liver metastasis (CRLM) organoids identified significant differences in spontaneous differentiation between those derived from primary CRC and those derived from liver metastases (LMs). Tumor stem cells within LM organoids possessed higher self-renewal potential than those in CRC organoids. Cell trajectory analyses indicated that tumor stem cells in CRC organoids tend to undergo differentiation, while mature cells in LM organoids exhibit dedifferentiation tendencies, underscoring the self-renewal capacity of tumor stem cells in LMs [258]. Additionally, one study has revealed distinct state transitions that CRC cells undergo during metastasis and how these changes influence adaptability and treatment response. Using single-cell sequencing to dissect the intrinsic plasticity and environmental adaptability of cells, it identified transcription factors that play critical roles in regulating gene transcription to name a fewprospero homeobox 1 (*PROX1*) [259].

Several key signaling pathways and proteins are involved in CRC pathogenesis. NLR family pyrin domain containing 12 (NLRP12) inhibits the Wnt/β-catenin pathway by interacting with serine/threonine kinase 38 (STK38), thereby suppressing CRC development and progression [260]. Research has demonstrated that *Nlrp12* deficiency increased CRC incidence and significantly elevated activation of the Wnt/β-catenin pathway, independent of the gut microbiota composition. This finding indicates that the NLRP12/STK38/GSK3β signaling axis may be a promising therapeutic target for CRC [260]. Further investigation revealed that NLRP12 inhibits the Wnt/β-catenin pathway by interacting with STK38, which acts as a crucial regulator in controlling the phosphorylation of GSK3β. Under normal conditions, GSK3β phosphorylates β-catenin, marking it for proteasomal degradation and maintaining low Wnt/β-catenin pathway activity. The interaction between NLRP12 and STK38 stabilizes the activity of GSK3β, allowing it to continuously phosphorylate β-catenin effectively, thereby preventing abnormal activation of the Wnt/β-catenin pathway. However, this inhibitory effect is lost in the absence of NLRP12, leading to β-catenin accumulating within the cell, excessive activation of the Wnt/β-catenin pathway, and promotion of CRC development [261].

Cetuximab is an anti-EGFR antibody commonly used to treat CRC without KRAS proto-oncogene GTPase (*KRAS*), NRAS proto-oncogene GTPase (*NRAS*), or B-Raf proto-oncogene serine/threonine kinase (*BRAF*) mutations [262]. However, despite the presence of these biomarkers, the response to cetuximab varies significantly, and predictive biomarkers remain limited. Previous preclinical studies have mainly relied on cell lines, which lack tumor complexity and often produce data that are difficult to translate due to the absence of the patient’s tumor microenvironment [263]. In order to address these limitations, studies have employed PDX models, which more closely mimic human tumor biology, to identify reliable predictive markers for cetuximab sensitivity in CRC. One study has made significant progress, demonstrating the value of using a large, well-characterized PDX collection and integrative multi-omics approaches in predicting cetuximab sensitivity [263]. Furthermore, research on personalized treatment for metastatic CRC has explored the feasibility and clinical efficacy of using tumor-derived organoids and in vitro drug sensitivity testing to provide customized treatment plans for patients [264]. These advances suggest that integrating advanced models and testing methods can enhance the precision of targeted cancer therapies.

Currently, organoids have become a critical tool in drug development, with their importance steadily increasing, particularly in replacing animal testing during drug screening. Related clinical trials are being vigorously pursued, demonstrating a thriving development trend. As shown in Table 2, organoids are making significant strides in clinical trials for drug screening as alternatives to animal testing. This shift not only enhances the efficiency and accuracy of drug screening but also reduces reliance on animal experiments, providing new opportunities and platforms for drug development. In breast cancer research, PDOs are regarded as promising models for overcoming the numerous limitations of cell lines and PDXs. However, many unanswered questions remain regarding the practical application of PDOs in breast cancer drug testing, particularly in treating patients with drug resistance and tumor recurrence.

**Table 2:** Clinical Trials of using organoids to replace animals for drug screening

Diseases	Research	Intervention/Treatment	Type	First Posted	NCT Number
Recurrent High Grade Glioma	PTCs-based Precision Treatment Strategy on Recurrent High-grade Gliomas	Drug: Receiving chemOtherapeutic or targeted Drugs recommended by molecular tumor board	Interventional	April 26, 2022	NCT05473923
Head and Neck Squamous Cell	PDO Based Drug Sensitive Test in R/M HNSCC	Drug Sensitive Test in Vitro	Observational	November 13, 2024	NCT06686342
Locally Advanced Thyroid Gland Carcinoma	Efficacy of Organoid-Based Drug Screening to Guide Treatment for Locally Advanced Thyroid Cancer	Drug: Anlotinib Drug: Lenvatinib Drug: Sorafenib Drug: Donafenib|Drug: Everolimus Drug: Apatinib Drug: Dabrafenib + Trametinib Drug: Cabozantinib Drug: Vandetanib|Drug: Entrectinib Drug: Pralsetinib Drug: Larotrectinib	Interventional	July 1, 2024	NCT06482086
Hepatocellular Carcinoma	Construction of a Recurrence Risk Prediction Model for Liver Resection Based on Drug Sensitivity of Patient-derived Hepatocellular Carcinoma Organoid	Drug: Adjuvant chemotherapy Device/Procedure: Adjuvant TACE (Transarterial Chemoembolization)	Observational	November 21, 2024	NCT06699524
Breast Cancer	Clinical Study on Drug Sensitivity Verification or Prediction of Therapy for Breast Cancer by Patient-Derived Organoid Model	Drug: Paclitaxel	Interventional	June 1, 2018	NCT03544047
Breast Cancer	Breast Cancer Treatment Based on Organ-like Culture	Drug: Trastuzumab Drug:Doxorubicin Hydrochloride Drug:Epirubicin hydrochloride Drug: Fluorouracil Drug: Paclitaxel Drug: Gemcitabine Drug: Cisplatin Drug: Recombinant Human Endostatin Drug: Pirarubicin hydrochloride Drug: Pyrrolidine Drug: Ixabepilone Drug: Tamoxifen citrate Drug: Vinorelbine tartrate Drug: Carboplatin Drug: Methotrexate Drug: Eribulin mesylate Drug: Toremifene citrate Drug: Anastrozole Drug: Letrozole Drug: Exemestane Drug: Fulvestrant Drug: Olaparib Drug: Bevacizumab Drug: Apatinib mesylate Drug: Pattozumab Drug: Capecitabine Drug: Ear particles Drug: Aidi Injection Drug: Cyclophosphamide	Observational	April 24, 2019	NCT03925233
Breast Cancer	Organoid Model Predictive of Response to ImmunOtherapies	Drug: ImmunOtherapy	Observational	October 16, 2023	NCT06084676
Advanced Breast Cancer	Organoid-based Functional Precision Therapy for Advanced Breast Cancer	Drug: Organoid-guided treatment Drug: Taxane Drug: Capecitabine Drug: Gemcitabine Drug: Vinorelbine Drug: Eribulin Drug: Anthracycline Drug: Carboplatin Drug: Utidelone Drug: Trastuzumab deruxtecan Drug: Sacituzumab govitecan	Interventional	October 26, 2023	NCT06102824
Lung Cancer	Organoids Predict Therapeutic Response in Patients With Multi-line Drug-resistant Lung Cancer	Drug: Antitumor therapy guided by organoid Drug sensitivity test	Interventional	January 3, 2023	NCT05669586
NSCLC Stage IV	Using Ex Vivo Tumoroids To Predict ImmunOtherapy Response In NSCLC	Drug: Standard of care immune checkpoint inhbitors	Observational	April 18, 2022	NCT05332925
Gastric Cancer	Gastric Cancer Organoids in the Screening of Neoadjuvant Drugs	Drug: Oxaliplatin	Observational	January 9, 2024	NCT06196554
Advanced Gastric Carcinoma	The Clinical Efficacy of Drug Sensitive Neoadjuvant ChemOtherapy Based on Organoid Versus Traditional Neoadjuvant ChemOtherapy in Advanced Gastric Cancer	Drug: PDO group|Drug: Traditional group	Observational	April 28, 2022	NCT05351398
Metastatic Colorectal Cancer	Study to Investigate Outcome of Individualized Treatment in Patients With Metastatic Colorectal Cancer	Drug: Alectinib Drug: Cetuximab Drug: Crizotinib Drug: Dasatinib Drug: Everolimus Drug: Encorafenib Drug: Gemcitabine Drug: Idelalisib Drug: Larotrectinib Drug: Methotrexate Drug: Palbociclib Drug: Panobinostat Drug: Pembrolizumab Drug: Petrozumab Drug: Trastuzumab Drug: Talazoparib Drug: Venetoclax	Interventional	February 13, 2023	NCT05725200
Pancreas Neoplasms	Organoid-driven ChemOtherapy Choice in Metastatic Pancreatic Cancer Patients	Drug: Standard chemOtherapy Drug: Organoid-guided treatment	Interventional	September 27, 2024	NCT06615830
Bladder Cancer	Guiding Instillation in Non Muscle-invasive Bladder Cancer Based on Drug Screens in Patient Derived Organoids	Drug: Epirubicin Drug: Mitomycin Drug: Gemcitabine Drug: Docetaxel	Interventional	August 27, 2021	NCT05024734
Bladder Cancer	Precise Neoadjuvant Chemoresection of Low Grade NMIBC	Drug: Epirubicin Drug: Mitomycin Drug: Gemcitabine Drug: Docetaxel	Interventional	January 26, 2024	NCT06227065
Abdominal Tumors	Patient-derived-organoid (PDO) Guided Versus Conventional Therapy for Advanced Inoperable Abdominal Tumors	Drug: PDO-guided treatment Drug: standard of care	Interventional	May 17, 2022	NCT05378048
Colorectal Cancer	Precision ChemOtherapy Based on Organoid Drug Sensitivity for Colorectal Cancer	Drug: Folfox, Folfiri or FOLFOXIRI regimens Drug: Folfoxor CapeOX regimens	Interventional	April 27, 2023	NCT05832398
Colorectal Cancer	Precision ChemOtherapy Based on Organoid Drug Sensitivity for Colorectal Cancer	Drug: Folfox, Folfiri or FOLFOXIRI regimens Drug: Folfoxor CapeOX regimens	Interventional	April 27, 2023	NCT05832398
Rare Tumour	Study of Precision Treatment for Rare Tumours in China Guided by PDO and NGS	Drug: Albumin-Bound Paclitaxel Drug: Epirubicin Drug: Gemcitabine Drug: Vinorelbine Drug: Cisplatin Drug: Irinotecan Drug: Fluorouracil Drug: Nivolumab Drug: Pembrolizumab Drug: Durvalumab Drug: Atezolizumab Drug:Sintilimab Drug: Tislelizumab Drug: Camrelizumab Drug: Toripalimab Drug: Serplulimab Drug: Adebrelimab Drug: Envafolimab Drug: Osimertinib Drug: Alectinib Drug: Vemurafenib Drug: Pamiparib Drug: Pyrotinib Drug: Imatinib Drug: Palbociclib Drug: Savolitinib Drug: Entrectinib Drug: Other	Interventional	November 18, 2024	NCT06692491
Neoadjuvant Therapy	The Clinical Efficacy of Drug Sensitive Neoadjuvant ChemOtherapy Based on Organoid Versus Traditional Neoadjuvant ChemOtherapy in Advanced Rectal Cancer	Drug: standard long-term therapy Drug: Folfoxand standard long-term radiOtherapy Drug: Folfiri and standard long-term radiOtherapy Drug: 5-FU and standard long-term radiOtherapy Drug: 5-FU and pembrolizumab and standard long-term radiOtherapy Drug: Other individualized treatments	Interventional	April 28, 2022	NCT05352165
Cystic Fibrosis	Clinical Trial to Evaluate the Efficacy and Safety of Dirocaftor/Posenacaftor/Nesolicaftor in Adults With CF	Drug: Diponecaftor Drug: Placebo	Interventional	June 21, 2024	NCT06468527

Data sources: clinical registration website “https://clinicaltrials.gov/”

In summary, single-cell sequencing technology has provided a notable high-resolution perspective for tumor organoid research, systematically revealing the high heterogeneity characteristics of major cancer types such as lung cancer, breast cancer, liver cancer, gastric cancer, and colorectal cancer. This technology precisely deciphers the gene expression profiles and signaling pathway networks within tumor cells and deeply describes the functional states and interactions of immune and stromal cells in the tumor microenvironment. By constructing disease-specific organoid biobanks and integrating multi-omics analysis, studies have successfully identified key signaling axes, including Wnt/β-catenin, HER2 and NLRP12/STK38/GSK3β, providing novel targets for the development of targeted therapies and combination drug strategies. Despite ongoing challenges in model standardization and clinical translation, the deep integration of tumor organoids with single-cell technologies is driving cancer research to leap from traditional population-level studies to single-cell precision, laying a solid technical foundation for drug screening, efficacy prediction, and the development of personalized treatment plans in the era of precision medicine.

## Application of single-cell sequencing in organoid-based disease models

The deep integration of single-cell sequencing and organoid technology has provided a platform for systematically dissecting disease mechanisms in highly biomimetic in vitro models. This chapter will focus on the revolutionary applications of this technological combination in the study of four major categories of diseases: neurodegenerative diseases, genetic diseases, infectious diseases, and metabolic diseases. Through multi-omics analysis at single-cell resolution, research can precisely identify disease-associated rare cell subpopulations, delineate cell lineage differentiation trajectories, and dynamically reveal key signaling pathway abnormalities and complex intercellular interaction networks. The following sections will sequentially elaborate on how this technology advances the understanding of specific diseases, for instance, Alzheimer's disease, Parkinson's disease, cystic fibrosis, SARS-CoV-2 infection, and diabetes, and demonstrate its immense potential in target discovery, drug screening, and the development of personalized treatment strategies.

### Neurodegeneration

#### Alzheimer’s disease

Alzheimer’s disease (AD) is primarily characterized by cognitive decline and behavioral changes and has a genetic predisposition. Rare mutations in genes including amyloid beta precursor protein (*APP*), presenilin 1 (*PSEN1*), and presenilin 2 (*PSEN2*) are associated with autosomal dominant AD, while polymorphisms in the apolipoprotein E (*APOE*) gene are associated with sporadic AD [265]. Pathologically, the accumulation of extracellular β-amyloid (Aβ) plaques and neurofibrillary tangles (NFTs) is the main feature of AD [266]. Traditional models often fail to capture AD’s complexity fully. Integrating organoid technology with single-cell sequencing provides new opportunities for exploring AD pathogenesis.

AD organoids excel in maintaining cellular heterogeneity and tissue specificity. Unlike traditional animal models, organoid models exhibit AD-related pathological features, Aβ plaques, and NFTs, within a human cellular system, offering a platform more reflective of human physiology and facilitating drug screening and efficacy assessment [267]. Single-cell sequencing is also employed in AD research to overcome the limitations of conventional bulk sequencing, which struggles with cellular heterogeneity in the CNS due to AD’s gradual and subtle onset. It has revealed key genes and therapeutic targets in AD pathogenesis at the single-cell level [268, 269].

**Fig. 4:**
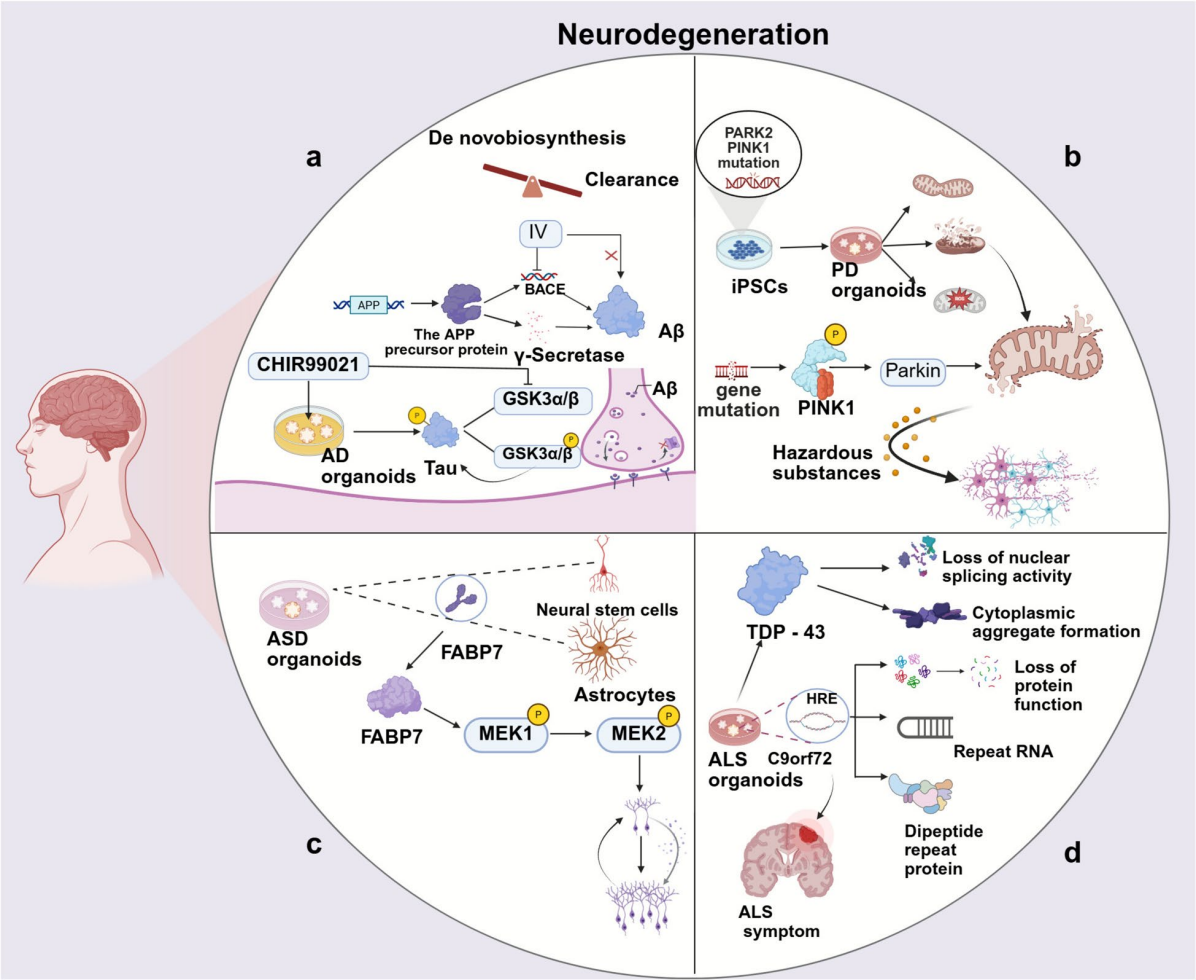
Molecular Mechanisms and Signaling Pathways in Neurodegenerative Diseases. **a** Aβ accumulation and tau hyperphosphorylation. In hiPSC-derived brain organoids exposed to serum, BACE expression increases, leading to Aβ accumulation and synaptic dysfunction. Tau hyperphosphorylation forms NFTs, with GSK3α/β playing a vital role. The GSK3α/β inhibitor CHIR99021 significantly reduced p-tau levels, indicating this pathway as a potential therapeutic target for AD. **b** Mutations in PARK2 and PINK1 in DNs reduce mitochondrial respiratory chain complex activity and disrupt membrane potential, causing energy metabolism imbalance and excessive ROS production, exacerbating oxidative stress. Disruption of the PINK1/parkin pathway hinders mitochondrial clearance, creating a vicious cycle leading to neuron death. Astrocytes and microglia exacerbate PD pathology through inflammatory responses. **c** The FABP7/MEK signaling pathway plays a key role in the abnormal differentiation of NSCs. Reduced FABP7 expression leads to aberrant MEK1/2 phosphorylation, triggering premature NSC differentiation, suggesting a close link between FABP7/MEK pathway abnormalities and ASD pathogenesis. **d** TDP-43 pathway abnormalities and C9orf72 HRE are critical. TDP-43 proteinopathy involves loss of nuclear splicing activity and cytoplasmic aggregation. Chronic oxidative stress induces TDP-43 mislocalization, SG formation, and splicing defects. C9orf72 HRE contributes to ALS through the loss of protein function, toxic repeat RNA, and DPRs, driving disease progression

The imbalance between Aβ production and clearance is central to AD pathogenesis. The *APP* gene encodes a precursor protein that generates Aβ via beta-secretase (BACE) and γ-secretase activity. The hiPSC-derived brain organoids used to model sporadic AD in vitro under serum exposure showed elevated *BACE* expression, leading to Aβ pathology. BACE inhibitor IV can reduce Aβ levels, and Aβ interacts with cell surface receptors, activating downstream pathways that affect neuronal synaptic function and survival [270]. Single-cell RNA sequencing of different hiPSC-derived brain organoids revealed that serum treatment does not significantly alter the growth and differentiation trajectories of cell subpopulations, including neurons, astrocytes, and neuroepithelial cells [270]. NFTs primarily comprise hyperphosphorylated microtubule-associated protein tau (MAPT/tau), with AD brain organoids exhibiting tau pathology. Various kinases and phosphatases regulate the phosphorylation of tau (p-tau), notably glycogen synthase kinase 3 alpha/beta (GSK3α/β), which plays a key role in its hyperphosphorylation [271, 272]. Under serum exposure, treatment with CHIR99021 (a GSK3α/β inhibitor) significantly reduced the levels of phosphorylated and total GSK3α/β and p-tau, indicating that serum exposure induced p-tau and that the GSK3α/β-tau signaling pathway is crucial in AD pathogenesis, identifying it as a potential therapeutic pathway (Fig. 4a). Another study indicated that *APOE* polymorphisms are closely associated with AD risk [273]. APOE is crucial in lipid metabolism, with the APOE4 variant exacerbating apoptosis and neurodegeneration in AD organoids [274] and the APOE3 Christchurch (APOE3ch) variant potentially having a protective effect [275]. APOE can bind to Aβ, influencing its metabolism and clearance, and modulates neuroinflammatory responses and neuronal survival, as APOE3ch enhances microglial phagocytosis to alleviate Aβ fibril aggregation and tau pathology [276]. Exploring APOE-related signaling pathways is significant for developing personalized therapeutic strategies in AD.

Integrating organoid technology with single-cell sequencing has led to remarkable breakthroughs in AD. One study utilized iPSC-derived hindbrain organoids to evaluate the treatment response of escitalopram oxalate, a selective serotonin reuptake inhibitor, on the neuropsychiatric symptoms of AD [277]. A systematic assessment using mouse and human organoid models revealed that semaglutide treatment decreased levels of Aβ, p-tau, and glial fibrillary acidic protein (GFAP) and increased oxytocin/neurophysin I prepropeptide (*OXT*) expression in the AD-brain organoid model [267]. Additionally, constructing AD organoid models and employing single-cell sequencing provided insights into cellular heterogeneity, allowing for a precise analysis of critical signaling pathways and proteins involved in AD, and identifying potential therapeutic targets.

Despite significant advantages, this field still faces challenges. Current brain organoids exhibit limited maturity, making it challenging to mimic the late-stage aging characteristics of AD fully. The absence or functional inadequacy of endogenous microglia restricts in-depth research into neuroimmune interactions. Technologically, key future innovations will focus on effectively capturing low-abundance yet critical electrically excitable neurons and integrating spatial transcriptomics data to correlate with the local microenvironment surrounding pathological protein deposits. Addressing these challenges will significantly enhance organoid models' predictive capabilities in deciphering AD's complex mechanisms and advancing therapeutic development.

#### Parkinson’s disease

Parkinson’s disease (PD) is the second most common neurodegenerative disorder after AD [278]. It is primarily characterized by the degeneration and death of dopaminergic neurons (DNs) in the substantia nigra, resulting in decreased dopamine levels in the striatum and significant motor control dysfunction [279]. Moreover, the aberrant accumulation of α-synuclein (SNCA) severely disrupts protein homeostasis and function within the nervous system [280]. Traditional models have limitations in elucidating PD pathogenesis. However, the advent of iPSC-based organoid models, midbrain organoid (MBO) and DN models [281], offers a comprehensive system. These organoids contain DNs and glial cells, such as astrocytes, facilitating the study of intercellular interactions. Single-cell sequencing further assists in analyzing cellular heterogeneity within organoids, uncovering potential therapeutic targets. PD organoids offer unique advantages over traditional models. They can more accurately recapitulate PD’s pathological features in vitro, for example SNCA accumulation and DN degeneration. They can also serve as high-throughput drug screening platforms, enabling rapid assessment of interventions targeting PD-related pathologies. For example, transplanting human MBOs into mouse models of PD improved motor dysfunction, highlighting organoids’ potential in cell therapy and evaluating drug efficacy [282].

Single-cell sequencing plays a pivotal role in PD organoids [283], precisely revealing cell subpopulation heterogeneity and enabling clear distinctions between DNs, astrocytes, and microglia [121]. The study found that within the substantia nigra pars compacta (SNpc), there are approximately 10 subtypes of dopamine-producing cells, but only the neuronal subpopulation expressing the *AGTR1* gene is selectively lost in PD patients [284]. This specific loss may be related to the inherent high genetic risk of this group and the abnormal response to stress signals targeting *TP53* and *NR2F2.* Furthermore, the technological system identified the activation state of pan-glial cells in PD, with microglia exhibiting increased mitochondrial activity and weakened immune function [285, 286], astrocytes displaying abnormalities in vesicle transport and mitochondrial function, and oligodendrocyte precursor cells also showing dysregulation of immune pathways, collectively constituting the cellular basis of neuroinflammation [287].

Abnormal SNCA aggregation is central to PD pathogenesis [288]. Under normal conditions, SNCA maintains homeostasis within neurons [289]. However, *SNCA* mutations, oxidative stress, and mitochondrial dysfunction cause SNCA to misfold into Lewy bodies. In MBOs without functional DJ1, glycation stress impairs lysosomal hydrolysis in astrocytes, resulting in the accumulation of SNCA oligomers and phosphorylated SNCA [290]. This aggregation impairs neuronal function and disrupts vesicular transport and organelle interactions, exacerbating neuronal damage and cell death, thus triggering a series of chain reactions that drive disease progression. Furthermore, mitochondrial dysfunction exacerbates PD progression. One study using DNs differentiated from iPSCs derived from a patient with PD in organoid models with *PARK2* and PTEN-induced kinase 1 (*PINK1*) mutations showed reduced mitochondrial respiratory chain complex activity, abnormal membrane potential leading to energy metabolism imbalance, and excessive reactive oxygen species (ROS) causing oxidative stress [291]. The PINK1/parkin signaling pathway regulates mitochondrial status. In response to mitochondrial damage, PINK1 phosphorylates parkin to induce mitophagy, clearing damaged mitochondria [292]. However, mutations can impair this pathway, hindering the removal of damaged mitochondria and releasing harmful substances that create a vicious cycle, ultimately leading to DN death in PD. Glial cells, including astrocytes and microglia, play a significant role in PD pathogenesis (Fig. 4b).

Research on organoid models has also clarified the non-autonomous role of glial cells in PD. One study using patient-derived iPSC-differentiated MBOs found that astrocyte dysfunction caused by *parkin* mutations may be a key factor in the non-cell-autonomous death of DNs [293]. These astrocytes secrete factors that interact with neurons, and when dysregulated, for instance via impaired lysosomal hydrolysis, they affect the neuronal microenvironment, promoting SNCA aggregation and neuronal damage. In PD organoids, activated microglia release inflammatory factors including tumor necrosis factor (TNF/TNF-α) and interleukin 1 beta (IL-1β), which activate signaling pathways like nuclear factor kappa B (NF-κB), exacerbating neuronal inflammatory response and damage [294]. Moreover, the interaction between glial cells and SNCA aggregates forms a complex pathogenic network, with each factor influencing the others and driving PD progression [295]. These studies demonstrate the complex interactions between glial cells and DNs in PD pathogenesis, highlighting the importance of organoids.

Currently, multi-omics integration has identified potential intervention targets, such as HSP90 and the ISR pathway, driving compounds like HSP90 inhibitors into preclinical research [296]. Despite challenges including limited sample availability and insufficient model standardization, the integration of organoids and single-cell technologies is progressively constructing more precise PD molecular networks, offering new hope for mechanism elucidation and therapeutic strategy development.

#### Autism spectrum disorder

Autism spectrum disorder (ASD) is a complex neurodevelopmental disorder characterized by impaired social interaction and communication, restricted interests, repetitive behaviors, and sensory abnormalities. As a childhood-onset condition, ASD has a complex genetic background involving hundreds of genes with diverse biological functions [297, 298], consequently, its pathogenesis remains incompletely elucidated. The emergence of brain organoid technology offers new approaches to studying ASD. Various ASD-related organoids can be generated from iPSCs. For example, single-gene mutation brain organoids have been developed for high-risk ASD genes, suppressor of variegation 4–20 homolog 1 (*SUV420H1*), AT-rich interaction domain 1B (*ARID1B*), and chromodomain helicase DNA binding protein 8 (*CHD8*) [297, 299–301]. These organoids can simulate cellular differentiation and proliferation events in brain development under specific culture conditions. Additionally, forebrain organoids derived from iPSCs from patients with specific gene mutations can be used to study the impact of such mutations on embryonic cortical development [302]. iPSC-derived brain organoids combined with single-cell sequencing technology provide a powerful tool for simulating human brain development in vitro and deciphering ASD-related cellular dynamics [303].

Single-cell sequencing of ASD brain organoids can distinguish various cell types, including neural stem cells (NSCs), excitatory neurons, inhibitory neurons, astrocytes, and microglia [304]. Characteristic changes in cell subpopulations are observed in organoids with mutations in different ASD risk genes. For example, in organoids derived from ARID1B gene mutations, single-cell analysis revealed dilated ventral radial glial cells and a significantly increased proportion transitioning to early oligodendrocyte precursor cells (OPCs), suggesting this gene may contribute to ASD pathogenesis by influencing glial lineage differentiation [303]. Similarly, using the CRISPR-human organoid-single-cell sequencing system, researchers systematically screened the effects of multiple ASD high-risk genes on cell fate during organoid development, identifying key roles of genes including TCF4 in vulnerable cell types like inhibitory neurons [305]. Functional maps at this cellular resolution level reveal the spatiotemporal expression effects of risk genes in specific cell types, as well as the remodeling of intercellular interaction networks during ASD pathogenesis. It provides fundamental cellular-level data that traditional animal models struggle to capture, thereby deepening our understanding of ASD pathogenesis.

Single-cell sequencing combined with organoid models has advanced our understanding of the specific mechanisms underlying ASD neurodevelopmental signaling pathways. One study identified the fatty acid binding protein 7 (FABP7)/MEK pathway as pivotal in the aberrant differentiation of NSCs in forebrain organoids derived from patients with ASD. FABP7 is a conserved protein that binds hydrophobic ligands and long-chain fatty acids and is highly expressed in NSCs and astrocytes within the brain [306]. It plays a crucial role in establishing radial glial fibers and maintaining neuroepithelial cells. In ASD, FABP7 is closely associated with the regulation of neurogenesis and cortical differentiation [307]. One study on ASD brain organoids observed reduced *FABP7* expression, leading to the altered phosphorylation status of downstream MEK1/2, which then triggers premature differentiation of NSCs [308]. Further investigation demonstrated that both *Fabp7* knockout and *Mek2* overexpression in mice resulted in autism-related repetitive, stereotyped behaviors and social deficits, strongly linking FABP7/MEK pathway abnormalities to ASD pathogenesis (Fig. 4c). Modulating the FABP7/MEK pathway can reverse inappropriate neural differentiation in ASD organoids, suggesting this pathway as a promising target for ASD therapy [306].

Subsequently, a study utilizing brain organoids to investigate autism (Pitt-Hopkins syndrome) found that specific mutations in transcription factor 4 (*TCF4*) can disrupt neuronal maturation. Gene therapy to restore gene function could rescue the disease phenotype [309]. Similarly, organoid models derived from patient-specific iPSCs, such as the CNTNAP2 mutation model, have successfully reproduced key pathological features, including cortical dysplasia.

Research further combined gene correction techniques to reverse-validate the pathogenic mechanisms of these genes, providing robust preclinical evidence for the development of personalized gene therapy. In summary, brain-like organoid models can highly simulate the complexity of human brain development and disease-specific phenotypes [303]. This makes them a powerful platform for high-throughput phenotypic characterization studies of disease susceptibility genes and screening potential therapeutic strategies in vitro, offering new solutions for elucidating disease mechanisms and precision medicine.

#### Amyotrophic lateral sclerosis

Amyotrophic lateral sclerosis (ALS) is a multifactorial neurodegenerative disease characterized by the progressive loss of motor neurons, leading to muscle weakness, atrophy, and, ultimately, death due to respiratory failure [310–312]. Organoid technology provides innovative models for ALS [313–315]. The iPSCs from patients with ALS can be used to construct various organoids, neuromuscular organoids (NMOs) that replicate ALS-related spinal neuromuscular pathologies [316], including skeletal muscle contraction defects, neuromuscular junction degeneration, and abnormal protein aggregation. Brain organoids subjected to mechanical injury to simulate traumatic brain injury (TBI) exhibit key pathological features, for instance neuronal death, excessive tau phosphorylation, and nuclear export of TAR DNA-binding protein 43 (TDP-43). Notably, organoids derived from C9orf72-SMCR8 complex subunit (C9orf72)-related patients exhibited more pronounced TDP-43 dysfunction [316]. Additionally, screening efforts have identified potassium inwardly-rectifying channel subfamily J member 2 (KCNJ2) as a potential therapeutic target [317]. Furthermore, brain organoids have been utilized to investigate changes in cellular architecture and connectivity and molecular pathological characteristics in patients with C9orf72-mediated ALS/frontotemporal dementia (FTD) [317, 318]. Single-cell sequencing helps analyze ALS-related cellular changes, exploring gene expression and interactions among various cell types [319].

Single-cell sequencing integrated with organoid models reveals cell type-specific pathological mechanisms in ALS. Abnormalities in TDP-43-associated pathways are crucial in ALS pathogenesis. TDP-43 proteinopathy is characterized by the loss of nuclear splicing activity and the formation of cytoplasmic aggregates [320]. Chronic oxidative stress can trigger mislocalization to the cytoplasm, stress granule (SG) formation, target gene splicing defects, and dysregulation of autophagy and senescence markers, indicating a close relationship with the autophagy pathway [317, 321]. The hexanucleotide repeat expansion (HRE) in *C9orf72* is common in ALS and FTD, with its pathogenesis involving the loss of protein function and the functional toxicity of repeat RNA or dipeptide repeat proteins (DPRs). Additionally, proteins associated with proteostasis imbalance, mutant superoxide dismutase 1 (SOD1) aggregates, involve valosin-containing protein (VCP), optineurin (OPTN), TANK binding kinase 1 (TBK1), and sequestosome 1 (SQSTM1) in protein homeostasis and degradation [322]; angiogenin (ANG) and FIG4 phosphoinositide 5-phosphatase (FIG4) in regulating protein synthesis; NIMA-related kinase 1 (NEK1) and TBK1 in protein degradation; and charged multivesicular body protein 2B (CHMP2B) and profilin 1 (PFN1) in protein transport (Fig. 4d). The abnormal expression of these proteins leads to cellular dysfunction and death.

In drug screening, single-cell data allows for in-depth analysis of differential drug responses across various cell types, enabling precise identification of drug targets for specific cell subpopulations or pathological processes. For example, drug screening in C9orf72 mutant ALS organoids revealed that compound GSK2606414 protects specific populations of diseased neurons [313]. Simultaneously, the screening identified genes, KCNJ2 as novel therapeutic targets for future treatments. This approach not only advances our understanding of the abnormal mechanisms underlying key pathways in ALS. For instance, simulating oxidative stress in organoids triggers TDP-43 cytoplasmic aggregation and stress granule formation, accompanied by autophagy-lysosomal dysfunction. It further reveals cellular heterogeneity and molecular mechanisms during disease progression, laying a theoretical foundation for developing innovative therapies [319].

Although single-cell sequencing combined with organoid models offers a novel perspective for deciphering multicellular circuit abnormalities in a physiologically relevant human context for ALS research, this system currently faces challenges related to insufficient model maturity and cell capture representativeness. Current neuromuscular organoids exhibit limitations in simulating mature neuromuscular junctions and long-term neurodegenerative processes. Nevertheless, this innovative model has been employed to deepen understanding of the development and dysfunction of corticospinal-muscular circuits, gradually establishing functional neural circuits applicable to studying human brain development and disease. This approach will drive the development of intervention strategies targeting key pathways and specific cell types, offering new hope for future personalized treatments and improved patient outcomes.

### Genetic disorders

#### Neurofibromatosis

Neurofibromatosis encompasses a group of autosomal dominant genetic disorders characterized by the formation of tumors in the nervous system due to mutations in specific genes [323, 324]. It is classified into types NF1, NF2, and NF3, with NF1 being the most common [325, 326]. In NF1, organoids derived from patient skin neurofibromas can recapitulate tumor cells’ structural and molecular characteristics in vitro [327]. Single-cell sequencing facilitated a deeper analysis of the organoids, enabling the precise identification of diverse cell types and subpopulations, chwann cells, fibroblasts, and immune cells, and elucidating their functional changes during disease progression [328]. Integrating organoid models with single-cell sequencing offers a comprehensive approach to uncovering the cellular and molecular basis of neurofibromatosis.

**Fig. 5:**
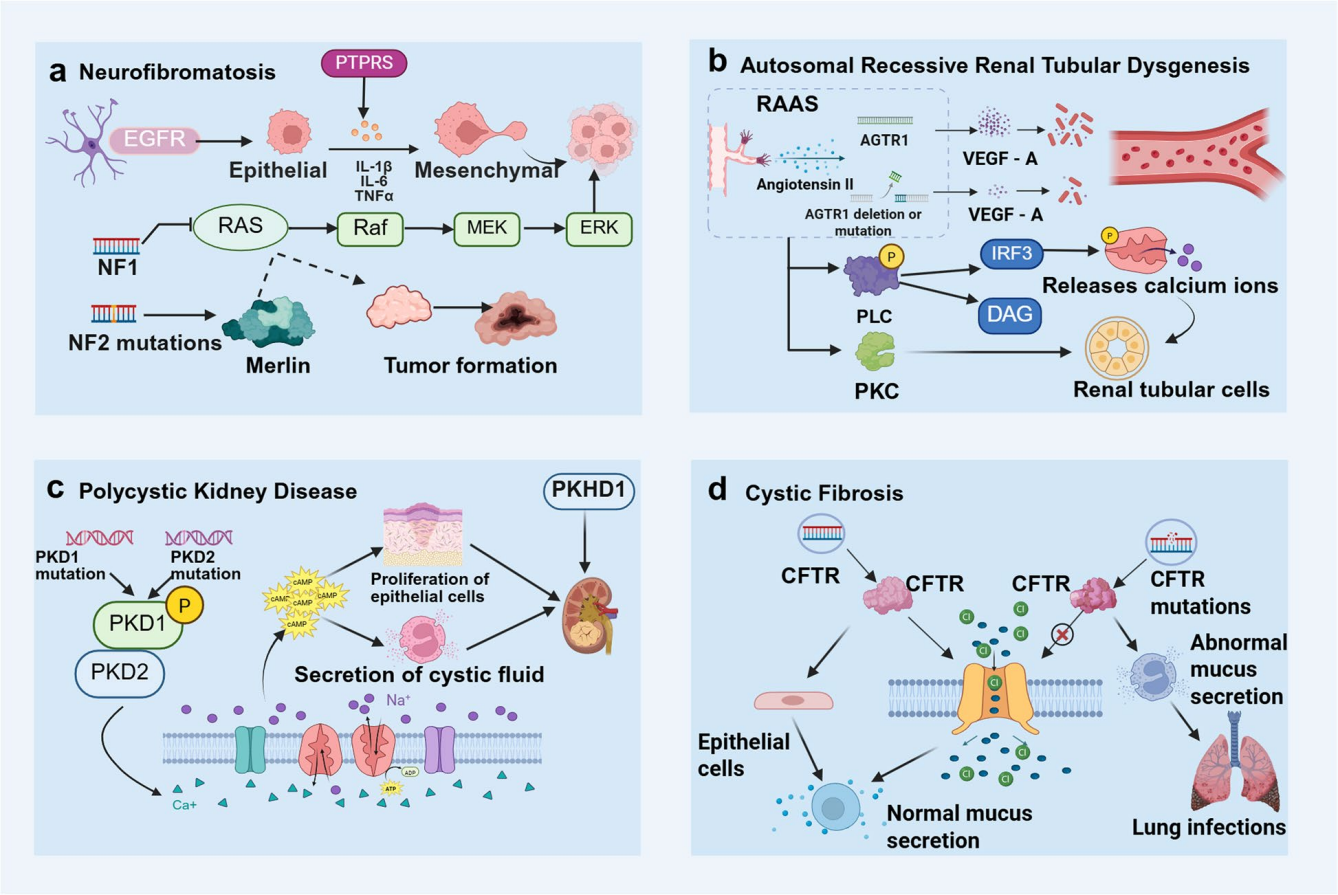
Molecular Mechanisms and Signaling Pathways in Genetic Diseases. **a** The role of NF1 and NF2 mutations in tumorigenesis. NF1 mutations lead to hyperactivation of the RAS signaling pathway, driving abnormal cell proliferation, which is fundamental in NF1-associated tumors. Reduced PTPRS expression enhances EGFR-mediated EMT, promoting cell migration and invasion in NF1-related tumors. NF2 mutations disrupt the RAS pathway and contribute to tumor development. **b** The impact of RAAS system gene mutations on AR-RTD pathogenesis. AGTR1 mutations or deletions decrease VEGFA expression, delaying angiogenesis and causing insufficient blood supply during critical nephron development, leading to dysplasia. These mutations also affect the PLC and PKC pathways, disrupting calcium signaling and impairing normal nephron cell proliferation and differentiation. **c** The role of PKD1, PKD2, and PKHD1 ciliary IPT domain containing fibrocystin/polyductin (PKHD1) mutations in PKD. Loss-of-function mutations in PKD1/PKD2 reduce calcium influx, increase cAMP accumulation, and promote cyst cell proliferation and fluid secretion. PKHD1 mutations affect the cellular signaling response, leading to cyst formation. Together, these mutations contribute to renal cystogenesis. **d** The core mechanism of CF. CFTR gene mutations result in dysfunctional CFTR protein, impairing chloride ion channel function, affecting epithelial fluid and electrolyte transport, and leading to abnormal mucus secretion

The integration of single-cell sequencing with organoid models reveals cell type-specific signaling activities and cellular state transitions in neurofibromas. In NF1, neurofibromin 1 (NF1) acts as a negative regulator of the RAS signaling pathway [329]. Mutations lead to hyperactivation of the RAS pathway, promoting abnormal cell proliferation, which is critical for NF1-associated tumorigenesis [330, 331]. Additionally, in the progression of NF1-associated plexiform neurofibromas, EGFR-mediated epithelial-mesenchymal transition (EMT) is regulated by the tumor suppressor protein tyrosine phosphatase receptor type S (PTPRS). Reduced *PTPRS* expression enhances EGFR-mediated EMT, facilitating tumor cell migration and invasion [332]. Moreover, mutations in NF2 moesin-ezrin-radixin-like (MERLIN) tumor suppressor (*NF2*/*merlin*) indirectly disrupt signaling pathways such as RAS, contributing to the formation of tumors like bilateral vestibular schwannomas (Fig. 5a). The PI3K/mechanistic target of rapamycin kinase (mTOR) signaling pathway is also affected by genetic mutations in both *NF1* and *NF2*, with its aberrant activation driving cell growth, proliferation, and survival, thereby playing a pivotal role in tumor development [333].

Applying single-cell sequencing in neurofibromatosis organoid models provides novel approaches and methodologies for drug screening, while also advancing the precision and preclinical translation of targeted intervention strategies. The synergistic activation of pathways including RAS/MAPK and PI3K/mTOR within specific cell populations, identified through single-cell data, provides a theoretical foundation for combination therapies [334]. Organoid models can further be used to evaluate the effects of MEK inhibitors or mTOR inhibitors on the proliferation and survival of different cell subpopulations, driving the development of personalized treatment strategies [327]. Furthermore, combining CRISPR screening with organoid-based drug sensitivity validation can identify novel synthetic lethal targets, expanding therapeutic options for treatment-resistant neurofibromas.

#### Autosomal recessive renal tubular dysgenesis

Autosomal recessive renal tubular dysgenesis (AR-RTD) is a severe hereditary kidney disorder characterized by significant maldevelopment of the proximal renal tubules [335]. It is caused by pathogenic mutations in genes related to the renin–angiotensin–aldosterone system (RAAS), leading to severely impaired renal function [336]. Traditional animal models struggle to accurately mimic the dynamic regulation of human-specific RAAS signaling pathways during renal tubular development. In contrast, kidney organoids derived from hPSCs, combined with single-cell sequencing, offer a novel humanized research platform for deciphering cell-type-specific signaling abnormalities and tubular developmental defects in the AR-RTD in vitro [336].

Mutations in RAAS system genes play a critical role in AR-RTD pathogenesis [337]. Angiotensin II binding to angiotensin II receptor type 1 (AGTR1) activates several intracellular signaling pathways, including those regulating vascular endothelial growth factor A (*VEGFA*) expression. VEGFA is a crucial angiogenic factor that promotes endothelial cell proliferation, migration, and tube formation. The deletion or mutation of *AGTR1* reduces the expression of *VEGFA*, leading to delayed angiogenesis. In critical phases of renal tubule development, inadequate angiogenesis reduces the blood supply around the proximal tubules, depriving them of essential nutrients and growth factors, leading to tubular dysplasia [338]. Additionally, mutations in RAAS system genes disrupt the secretion of intracellular signaling molecules including phospholipase C (PLC) and protein kinase C (PKC), disturbing the normal developmental program of renal tubule cells [337]. For example, PLC activation produces inositol trisphosphate (IP3) and diacylglycerol (DAG), with IP3 facilitating calcium release from intracellular stores (Fig. 5b). As secondary messengers, calcium ions are involved in many cellular physiological processes. Mutations affecting PLC activation lead to abnormal calcium signaling, disrupting the proliferation and differentiation of renal tubule cells [339].

Research based on single-cell data has identified weakened VEGF signaling and delayed vascular development, suggesting that pro-angiogenic drugs may serve as potential therapeutic strategies [340]. Additionally, dysfunction in the PLC/IP3/DAG pathway within renal tubular progenitor cells provides a theoretical basis for regulating intracellular calcium signaling to promote tubular maturation [341]. Following CRISPR/Cas9-mediated correction of the disease-causing gene, improved tubular structure was observed in organoid models [208]. Combined with single-cell sequencing to assess restored gene expression, this provides preclinical evidence for the feasibility and efficacy of gene therapy [211].

Current kidney organoids still have limitations in simulating the structure of mature nephrons and constructing vascular networks, which affects the complete recreation of the RAAS system's systemic regulatory functions [342]. Furthermore, discrepancies between the proportions of organoid cell types and those in developing kidneys may introduce interpretive biases. Incorporating vascularization strategies or organ-on-a-chip technologies, combined with spatial transcriptomics analysis, holds promise for more authentically simulating the developmental process of the tubulo-microvascular unit at both structural and functional levels, thereby enhancing the physiological relevance of research [209].

#### Polycystic kidney disease

Polycystic Kidney Disease (PKD) is a common hereditary kidney disorder characterized by progressively enlarging cysts in both kidneys. Its primary forms are autosomal dominant PKD (ADPKD) and autosomal recessive PKD (ARPKD) [343]. Most patients ultimately progress to end-stage renal disease, and effective treatments are currently lacking. Organoid technology provides a crucial model for PKD [344, 345]. For example, organoids with gene edits targeting polycystin-1 transient receptor potential channel interacting (PKD1/PC1) and polycystin-2 transient receptor potential cation channel (PKD2/PC2) can simulate cyst formation and related disease characteristics[346, 347]. Single-cell sequencing facilitates in-depth analysis, revealing changes in gene expression and chromatin accessibility among different cell types within PKD organoids. This technology has uncovered metabolic abnormalities, dysregulation of glycolysis and autophagy pathways, and altered cell interactions during cyst formation, offering critical insights into PKD.

In PKD1 or PKD2 mutant organoids, single-cell sequencing can identify cell populations including cyst wall epithelial cells, fibroblasts, and endothelial cells. The pathogenesis of PKD is closely linked to loss-of-function mutations in *PKD1* and *PKD2* [348]. Dysregulation of intracellular calcium signaling affects cAMP metabolism, promoting cyst formation. Calcium channel blockers can induce cyst formation and upregulate cAMP, indicating that calcium homeostasis and cAMP metabolism are critical interacting factors in PKD progression. The primary cilia-autophagy signaling axis is also involved [349]. In ARPKD kidney organoids, overexpressing autophagy-related 5 (*ATG5*) or knocking out the primary cilia-associated gene kinesin family member 3 A (*KIF3A*) activates autophagy and suppresses cyst formation, with cilia ablation accompanied by upregulated autophagy [350]. Additionally, eukaryotic ribosomal selective glycosides (ERSGs) can restore protein expression by inducing readthrough of nonsense mutations in *PC1* and *PC2* [346], reducing cyst formation, which highlights the significance of translational anomalies in PKD pathogenesis (Fig. 5c).

The study reports that loss of function in the PC1/PC2 complex disrupts intracellular calcium homeostasis, leading to cAMP accumulation and promoting cyst fluid secretion and epithelial proliferation. ERSG-like drugs identified through single-cell data analysis can restore partial PC1/PC2 expression by promoting the correction of nonsense mutations, thereby significantly inhibiting cyst formation in organoids [346]. Combining CRISPR gene correction with single-cell assessment enables further validation of pathogenic gene functions and optimization of gene therapy strategies, providing a basis for personalized treatment.

Pre-kidney organoids currently have limitations in simulating late-stage fibrotic microenvironments or cyst wall-stromal interactions. Low-frequency cellular events during cyst formation may also be overlooked due to insufficient sequencing coverage. By integrating spatial transcriptomics with live-cell dynamic imaging, it is possible to track cyst development trajectories while preserving structural information, thereby enhancing our understanding of disease initiation and progression mechanisms. Furthermore, the introduction of engineered culture systems, fluid shear stress stimulation devices holds promise for better simulating the impact of in vivo mechanical microenvironments on cyst development.

#### Cystic fibrosis

Cystic fibrosis (CF) is a common autosomal recessive disorder caused by mutations in the CF transmembrane conductance regulator (*CFTR*) gene, leading to dysfunctional CFTR protein and impaired chloride ion channels on epithelial cell membranes. This results in disrupted fluid and electrolyte balance, causing mucus accumulation in organs, the lungs, pancreas, and intestines, ultimately leading to organ failure and high mortality [351]. Organoid models derived from patient cells, rectal and airway epithelial organoids, can replicate some pathological features of CF [352]. Traditional cell models struggle to replicate the complex structure and function of organ-specific epithelia in the human body. However, patient-derived organoids derived from the rectum, airways, and pancreas, combined with single-cell sequencing technology, provide a highly physiologically relevant platform for studying the cell-specific effects of CFTR dysfunction and cross-organ pathological mechanisms in vitro [353].

The integrated application of single-cell sequencing and CF organoids enables high-resolution analysis of dynamic changes in cellular subpopulations during pathological processes, overcoming limitations of traditional research methods [354]. For instance, in CF airway organoid studies, single-cell sequencing precisely identified abnormal activation of goblet cell subpopulations driven by the F508del mutation. These cells specifically overexpressed the mucus gene MUC5AC and the inflammatory cytokine IL-8. Such fine-grained cell-type-specific alterations are often averaged out and obscured in traditional bulk sequencing, preventing direct linkage to clinically observed pathological features like excessive mucus secretion [355]. More importantly, this integrated approach revealed the dysregulated role of ubiquitin ligase RFFL in specific epithelial cell subpopulations. Under treatment with CFTR modulators like ivacaftor, RFFL persistently mediated the degradation of mutant CFTR proteins. This discovery elucidates the molecular mechanism underlying poor response to existing therapies in some patients and provides experimental rationale for developing combination therapies targeting RFFL.

The core pathology of CF is mutations in the *CFTR* gene, which result in dysfunctional CFTR protein, impairing chloride ion channel function. Under normal conditions, CFTR regulates fluid and electrolyte transport in epithelial cells, ensuring proper mucus secretion and clearance [356, 357]. Mutated CFTR leads to abnormal mucus secretion, causing recurrent infections and inflammation in the lungs, affecting enzyme secretion in the pancreas, and disrupting nutrient absorption in the intestines. Furthermore, the ring finger and FYVE-like domain containing E3 ubiquitin-protein ligase (RFFL) influences the functionality of F508del CFTR by removing it from the cell surface, even in the presence of CF medications, thereby reducing drug efficacy (Fig. 5d). This observation suggests that abnormalities in protein degradation pathways also play a significant role in CF pathogenesis [358].

In drug screening, integrating single-cell sequencing can provide an in-depth analysis of gene expression changes across different cell types after drug treatment, aiding in identifying drug targets specific to particular cell subpopulations or pathological processes. For example, examining single-cell data from CF organoids before and after CFTR modulator treatment could reveal new aspects of drug action and potential targets, optimizing therapeutic strategies [359, 360]. In assessing the efficacy of gene therapy, single-cell sequencing can evaluate the impact of gene editing on cell function and gene expression in CF organoid models, providing precise assessments of therapeutic outcomes. While the application of single-cell sequencing in CF organoids is currently limited, technological advances are expected to significantly support precision medicine for CF, facilitating the development of more effective treatments and improving patient prognosis.

### Infectious diseases

#### SARS-CoV-2 infection

Infection with severe acute respiratory syndrome-coronavirus 2 (SARS-CoV-2), responsible for the global COVID-19 pandemic, has profoundly impacted human health, socioeconomics, and societal order [361]. Organoid technology has demonstrated unique advantages in COVID-19 research [362, 363], overcoming the limitations of traditional cell lines and animal models to provide novel insights into cell-specific mechanisms of virus-host interactions. For instances, nasal epithelial organoids derived from primary human nasal epithelial cells cultured at the air–liquid interface contain diverse cell types including ciliated cells, goblet cells, and basal cells. These organoids mimic the physiological structure and function of the upper respiratory tract, providing a highly physiologically relevant model system for investigating SARS-CoV-2 infection mechanisms and host responses.

Single-cell sequencing technology has deepened our understanding of viral infection pathways from a cell-specific perspective. Studies have revealed that in nasal epithelial organoids, single-cell sequencing analysis showed ACE2 and TMPRSS2 localized on the organoids' cilia. This suggests SARS-CoV-2 may breach the airway barrier and infect cells via this pathway. Concurrently, changes in microvilli following viral infection were observed [364]. Alveolar organoids, composed of type I and type II alveolar epithelial cells and mesenchymal cells, have been shown to be permissive for SARS-CoV-2 infection and capable of reproducing host immune response characteristics post-infection [363, 365]. Single-cell sequencing technologies have further deepened our understanding of viral infection. For instance, Single-cell sequencing has resolved the response of lung cells to SARS-CoV-2 infection at the single-cell level, revealing infection dynamics, cellular heterogeneity, and non-classical infection pathways. It has also clarified the crucial role of pulmonary surfactant protein B in host immune responses and apoptosis [366]. The integration of these approaches facilitates a more comprehensive elucidation of the cellular and molecular mechanisms underlying SARS-CoV-2 infection.

**Fig. 6:**
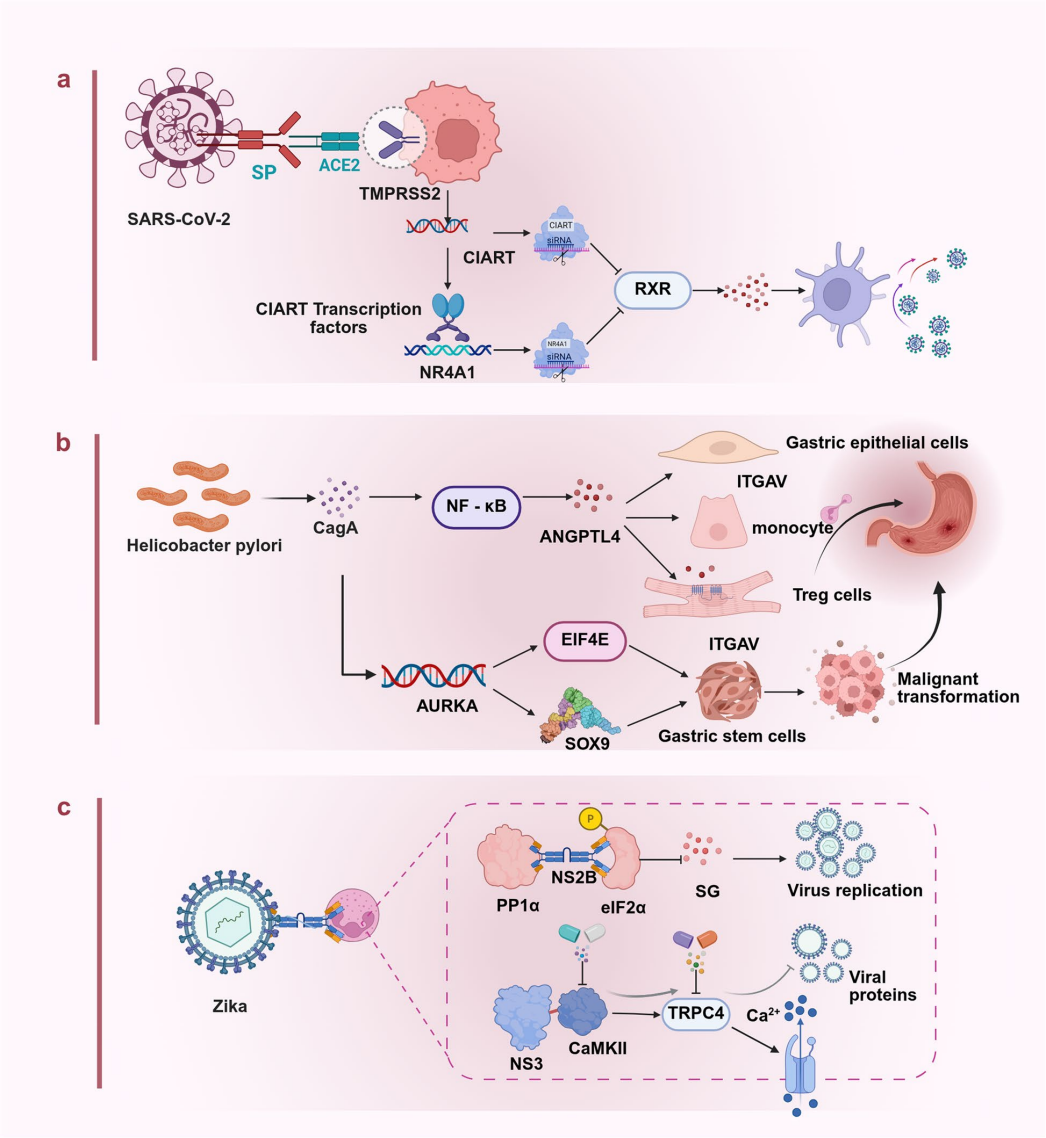
The Molecular Mechanisms and Signaling Pathways of Infectious Diseases. **a** SARS-CoV-2 infects host cells through its SP binding to ACE2, facilitated by TMPRSS2 for cell fusion. CIART influences NR4A1 expression, affecting cellular susceptibility. Knocking out CIART and NR4A1 disrupts the RXR signaling pathway linked to fatty acid metabolism, impacting viral infection. RXR inhibitors significantly reduce infection. **b** H. pylori protein CagA activates the NF-κB pathway, upregulating IL8 and ANGPTL4, promoting inflammation and colonization. ANGPTL4 interacts with ITGAV, regulating CCL5 secretion and Treg cell proliferation. AURKA enhances SOX9 expression, driving the malignant transformation of gastric stem cells and tumorigenesis. **c** Zika virus protein NS2B facilitates the interaction between PP1α and eIF2α, inhibiting SG formation and enhancing replication. Viral protein NS3 increases TRPC4 expression via CaMKII, enhancing calcium influx, thus identifying it as a potential therapeutic target

The SARS-CoV-2 infection process involves multiple key signaling pathways and proteins. The viral spike protein (SP) binds to the ACE2 receptor on the surface of host cells, mediated by host TMPRSS2, which facilitates viral fusion and entry into the cells, marking the critical initial step of infection [361]. Within host cells, the transcription factor circadian associated repressor of transcription (CIART) plays an essential role in the SARS-CoV-2 infection process [367]. CIART binds to the promoter region of nuclear receptor subfamily 4 group A member 1 (*NR4A1*), influencing its chromatin accessibility and regulating its expression. Notably, knocking out *NR4A1* reduces the susceptibility of cells to SARS-CoV-2 infection [366]. Further experiments revealed that knocking out *CIART* suppressed the retinoid X receptor (RXR) signaling pathway, and knocking out *NR4A1* also downregulated genes associated with RXR signaling. Small-molecule inhibitors targeting the RXR pathway also significantly inhibited SARS-CoV-2 infection [368]. This pathway is closely linked to fatty acid metabolism, and its dysregulation affects the viral infection process (Fig. 6a). The study also validated the cell-specific mechanism by which existing drugs, imatinib exert therapeutic effects by interfering with viral entry pathways, providing crucial evidence for developing targeted therapeutic strategies.

Applying single-cell sequencing in organoid models of SARS-CoV-2 infection has led to significant breakthroughs [369]. In drug screening, single-cell data enables the precise identification of potential drug targets by revealing gene expression changes before and after infection, identifying genes and guiding the development of targeted therapies [370]. This approach has led to the discovery of drugs like imatinib, which can inhibit viral entry or replication [371]. In gene therapy, gene editing technologies can manipulate relevant genes in organoid models, knocking out phospholipid scramblase 1 (*PLSCR1*) to assess changes in susceptibility to viral infection, thereby informing the optimization of gene therapy strategies [372]. Combining single-cell sequencing with organoid models also allows real-time monitoring of cellular changes during treatment, enhancing understanding of therapeutic effects, improving efficacy and safety, and advancing precision medicine for SARS-CoV-2 infection.

#### *Helicobacter pylori* infection

*H. pylori* infection is a global gastric bacterial disease closely associated with the development of chronic gastritis, gastric ulcers, and gastric cancer (GC), imposing a significant health burden [373]. Historically, in vivo studies of *H. pylori* pathogenesis have primarily relied on mouse and gerbil models. However, regional and population-specific variations in *H. pylori* strains and differences in bacterial virulence, immune response, and microbiota between rodent models and humans present challenges in translating findings from animal models to human therapeutic contexts [374, 375]. Ethical and practical constraints further restrict vaccine and therapeutic testing in primate models. While challenge models of *H. pylori* infection have been established in human volunteers, these have been limited. While mice share a relatively close genetic similarity with humans, differences in genetic background can result in variable outcomes [376, 377]. Few knockout and transgenic mouse models progress to cancer and do not advance to the metaplasia stage, limiting their application in GC research [378].

Organoid technology has been instrumental in studying *H. pylori* infection. Gastric organoids, which replicate the physiological structure and function of the gastric mucosa, provide an ideal research model. *H. pylori* has been shown to infect various cell types within gastric organoids, pit cells and gastric gland stem cells, with a distinct preference for pit cells, which is associated with the bacterium’s chemotaxis toward host cell-released urea and cell size [379]. Furthermore, organoids have revealed the impact of *H. pylori* infection on gastric stem cell proliferation and differentiation, triggering the secretion of R-spondin 3 (RSPO3) by gastric gland myofibroblasts, affecting stem cell proliferation [380]. Single-cell sequencing can provide an in-depth analysis of the infection process from a cellular heterogeneity perspective. Sequencing gastric cells from different origins can reveal variations in gene expression and intercellular interactions during infection. Research indicates that 3D gastric organoids mainly comprise cells from glandular regions, while 2D monolayers predominantly comprise pit cells and their progenitors, with *H. pylori* preferentially binding to highly differentiated pit cell subpopulations [379]. Additionally, single-cell sequencing has uncovered that *H. pylori* infection activates the NF-κB pathway to induce inflammatory responses in gastric epithelial organoids [381]. Integrating organoids with single-cell sequencing offers a comprehensive and precise perspective on *H. pylori* infection.

The pathogenesis of *H. pylori* infection involves multiple key signaling pathways and protein interactions. The NF-κB signaling pathway is central among inflammation-related pathways [382]. *H. pylori* virulence factors, cytotoxin-associated gene A (CagA), activate the NF-κB pathway, leading to the upregulation of proinflammatory cytokines like interleukin 8 (IL8), which attract neutrophils and trigger inflammatory responses. This process is evident in gastric epithelial organoid infections and GC progression [381]. The angiopoietin-like 4 (ANGPTL4)-related pathway is also involved. *H. pylori*, dependent on the CagA virulence factor, activates the NF-κB pathway in gastric epithelial cells, promoting ANGPTL4 expression and secretion. ANGPTL4 interacts with integrin subunit alpha V (ITGAV) on gastric epithelial cells, monocytes, and regulatory T (Treg) cells, affecting neutrophil infiltration, C–C motif chemokine ligand 5 (CCL5) production by monocytes, and Treg cell proliferation, thereby promoting *H. pylori* colonization and gastritis progression [383]. The aurora kinase A (AURKA)/SRY-box transcription factor 9 (SOX9) signaling axis is pivotal in *H. pylori*-induced gastric tumorigenesis. *H. pylori* infection induces *AURKA* expression, which increases eukaryotic translation initiation factor 4E (EIF4E)- and cap-dependent translation, elevating *SOX9* polycistronic RNA levels and influencing the malignant transformation of gastric stem cells [384]. Additionally, the RSPO3 pathway affects gastric stem cell differentiation and glandular homeostasis during *H. pylori* infection (Fig. 6b). Chronic *H. pylori* infection maintains R-spondin-driven regeneration, potentially leading to glandular hyperproliferation and precancerous metaplasia development [379].

In drug screening, single-cell data facilitates the identification of potential drug targets. Conducting single-cell sequencing of cells before and after *H. pylori* infection can precisely identify genes that exhibit significant changes during infection, which may be associated with key pathological processes including bacterial colonization, inflammatory responses, or abnormal cell proliferation [379]. This promising approach may promote targeted and effective treatment strategies for *H. pylori* infection.

#### Zika virus infection

Infection with the Zika virus, which is primarily transmitted through *Aedes* mosquito bites, is an infectious disease associated with conditions including congenital Zika syndrome, which includes neurological defects like microcephaly, and Guillain–Barré syndrome [381, 385]. A study using brain organoids demonstrated that infection with the Zika virus significantly perturbs organoid cultures, reducing the number of neural progenitor cells and causing growth defects. These findings correlate with the neurological abnormalities observed in newborns following Zika infection, highlighting the value of brain organoids in studying the neurotropism of the Zika virus [386]. Research using trophoblast organoids has identified undifferentiated human trophoblast stem cells (hTSCs) as primary targets for the Zika virus within the placenta. The Zika virus can infect these trophoblast organoids, disrupting their structure, inhibiting syncytialization, and consequently affecting placental development [387–390].

Single-cell sequencing has been used to analyze transcriptomic changes in placental trophoblast organoids before and after Zika virus infection. This approach has clarified the cellular composition within the organoids, identifying distinct subpopulations including hTSCs, cytotrophoblasts (CTBs), CTB fusion cells, and syncytiotrophoblasts (STBs) while elucidating developmental trajectories. Susceptibility to the Zika virus was shown to decrease as trophoblasts differentiate. Additionally, some classical antiviral interferons (IFNs) and interferon-stimulated genes (ISGs) are activated in hTSCs post-infection, potentially affecting trophoblast function, inhibiting syncytialization [391]. Moreover, single-cell sequencing can precisely differentiate subpopulation functions during Zika virus infection. For example, yolk sac-derived primitive macrophages facilitate fetal brain infection, recruited monocytes contribute to non-productive neuroinflammation, and differentiated microglia in the brain exhibit protective roles in fetal mononuclear phagocytes [392]. Therefore, it offers a high-resolution perspective on fetal immune cells in congenital Zika virus infection.

The pathogenesis of Zika virus infection involves interactions among several key signaling pathways and proteins. The viral nonstructural protein 2B (NS2B)/protein phosphatase 1 catalytic subunit alpha (PP1α)/eukaryotic translation initiation factor 2 alpha (eIF2α) axis plays a critical role in viral replication. NS2B acts as a scaffold to mediate the interaction between PP1α and eIF2α, promoting eIF2α dephosphorylation and inhibiting SG formation, thus facilitating viral replication. The NS2B/PP1α complex exhibits high stability, whereas the NS2B-V35A mutant fails to inhibit SG formation, reducing viral replication [393]. Additionally, Zika virus infection increases transient receptor potential cation channel subfamily C member 4 (*TRPC4*) expression through the interaction of the viral structural protein 3 (NS3) with Ca^2+^/calmodulin-dependent protein kinase II (CaMKII), enhancing calcium influx. Inhibiting CaMKII or TRPC4 reduces viral protein production, suggesting TRPC4 or CaMKII as potential therapeutic targets [394]. During endoplasmic reticulum (ER) stress and autophagy, the viral nonstructural protein 2 A (NS2A) mediates the specific degradation of the ER autophagy receptor family with sequence similarity 134, member B (Fig. 6c).

This process is regulated by the ubiquitination of NS2A through the E3 ligase autocrine motility factor receptor (AMFR), inhibiting ER autophagy and exacerbating microcephaly symptoms and viral pathogenicity [395]. Regarding immune-related pathways and cellular functions, different subpopulations of fetal mononuclear phagocytes exhibit distinct functions, with their transcriptional profiles revealed by single-cell sequencing [392]. The investigation of Zika virus infection utilizing brain organoids, placental trophoblast organoids, and single-cell sequencing has gradually revealed the virus’s pathogenic process.

### Metabolic diseases

#### Diabetes

Diabetes is a common chronic metabolic disease, primarily classified into type 1 (insulin-dependent) and type 2 (non-insulin-dependent). Organoid and single-cell sequencing technologies provide powerful tools for in-depth studies of diabetes. Research on pancreatic islet organoids helps elucidate islet cell function and the pathogenesis of diabetes. For example, the cultivation and analysis of pancreatic islet organoids can simulate the physiological activities of islet cells [396]. In the field of vascular organoids, a study has shown that vascular organoids derived from donors with diabetes exhibit functional impairment and altered cellular heterogeneity. Single-cell sequencing has clarified molecular differences between vascular cell types and subpopulations, providing a basis for research into diabetic vascular complications [397]. Intestinal organoid models have been used to study the relationship between diabetes and intestinal barrier function, revealing that hyperglycemia impairs the intestinal barrier. Furthermore, single-cell sequencing showed that small-molecule hyaluronic acid (HA35) can protect barrier function [398].Pancreatic organoids, vascular organoids, and intestinal organoids respectively mimic key pathological pathways associated with diabetes, providing highly bio-inspired model systems for investigating the multi-systemic effects of metabolic disorders.

**Fig. 7:**
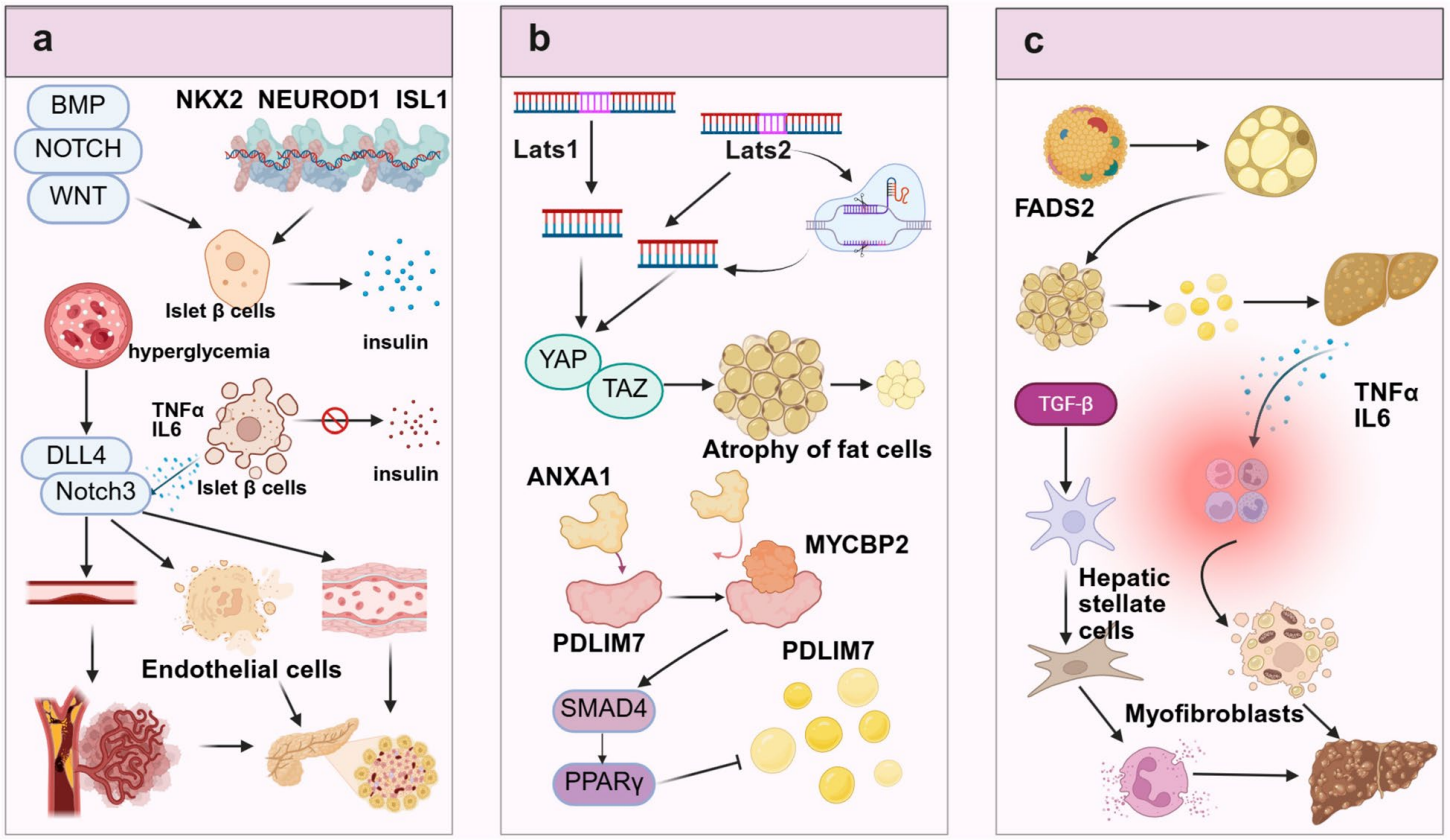
Molecular Mechanisms and Signaling Pathways in Metabolic Diseases. **a** The molecular mechanisms of diabetes: Pancreatic β-cell function is regulated by pathways including BMP, NOTCH, and WNT, as well as transcription factors such as NKX2, NEUROD1, and ISL1. Their dysregulation leads to insufficient insulin secretion. Hyperglycemia and inflammatory factors (TNF-α and IL6) activate the DLL4/NOTCH3 pathway, resulting in vascular basement membrane thickening, endothelial dysfunction, reduced capillary density, and vascular leakage. **b** The role of the Hippo-YAP/TAZ signaling pathway in obesity. Loss of LATS1/2 activates YAP/TAZ, causing adipocyte atrophy and energy imbalance. ANXA1 regulates SMAD4 degradation and PPARG transcription to inhibit adipogenesis, and its absence exacerbates obesity and metabolic disorders. **c **The pathogenesis of NAFLD. FADS2 promotes hepatic steatosis by regulating lipid metabolism. TNF activates inflammation, while TGF-β induces hepatic stellate cells to transdifferentiate into myofibroblasts, releasing extracellular matrix and forming fibrotic scars. These processes drive the progression of NAFLD from lipid metabolism disorders to inflammation and fibrosis

The pathogenesis of diabetes involves multiple key signaling pathways. Regarding pancreatic β-cell function, BMPs, NOTCHs, WNTs, and transcription factors such as NKX2, neuronal differentiation 1 (NEUROD1), and ISL LIM homeobox 1 (ISL1) are critical for islet development and insulin secretion regulation. Dysregulation of these factors can lead to abnormal insulin secretion. In diabetic vascular complications, the γ-secretase target notch receptor 3 (NOTCH3) and its ligand delta-like canonical Notch ligand 4 (DLL4) are central mediators of vascular basement membrane thickening, influenced by hyperglycemia and inflammatory factors [397, 399]. Regarding intestinal barrier function, tight junction proteins zona occludens 1 (ZO-1), OCLN, and claudin 1 (CLDN1) are crucial for maintaining barrier integrity, with hyperglycemia altering their expression. HA35 modulates these proteins through layilin (LAYN) to maintain cellular homeostasis (Fig. 7a). In virus-induced diabetes, pro-inflammatory macrophages secrete IL1β, which triggers pyroptosis in β-cells via the TNF superfamily member 12 (TNFSF12)/TNF receptor superfamily member 12 A (TNFRSF12A) pathway, altering related gene expression [400].

The single-cell sequencing of organoid models holds significant promise for diabetes research. In drug screening, single-cell transcriptomic data enable precise identification of key genes associated with abnormal insulin secretion, vascular lesions, and intestinal barrier dysfunction, providing candidate targets for targeted drug development. For instance, analyzing single-cell data from vascular organoids derived from diabetic donors revealed abnormal activation of the DLL4-Notch3 signaling pathway in specific endothelial cell subpopulations, opening new avenues for developing treatments targeting vascular complications [397, 401]. Moreover, gene editing can be used to manipulate key genes within organoid models, assessing their impact on cellular function restoration and informing gene therapy strategies. For example, the Wolframin ER transmembrane glycoprotein (*WFS1*) gene related to pancreatic β-cell function could be evaluated to improve insulin secretion [402].

#### Obesity

Obesity is a multifactorial, chronic metabolic disorder characterized by excessive fat accumulation, resulting in various physiological and metabolic disturbances. It is closely associated with severe complications of cardiovascular disease, diabetes, and cancer, posing a significant threat to human health [403]. Organoids can provide crucial models for obesity. For example, the stromal vascular fraction (SVF) from adipose tissue can form various adipose organoids, replicating the complex structure and function of adipose tissue and facilitating studies on adipogenesis and inter- rgan interactions [404]. Adipocyte organoid models derived from hPSCs can be differentiated using specific transcription factors, facilitating the exploration of obesity-related metabolic diseases. Additionally, brain organoids are used to study the link between obesity and depression. For example, the CRISPR/Cas9 system can create *WFS1*-knockout brain organoids from hESCs, revealing the pathogenesis of the vicious obesity-depression cycle [402]. Single-cell sequencing technology can track the differentiation trajectories of specific neural cell lineages in response to obesity-related stimuli. This discovery is challenging to identify in traditional batch sequencing.

The Hippo-Yes-associated protein (YAP)/transcriptional co-activator with PDZ-binding motif (TAZ) signaling pathway is critical in the pathogenesis of obesity. Adipocyte-specific knockout of large tumor suppressor kinases 1 (*Lats1*) and 2 (*Lats2*) activated YAP/TAZ, resulting in adipocyte atrophy [405], which affects adipose tissue quality and systemic energy balance. This effect involves the YAP/TAZ-TEA domain transcription factor (TEAD) axis regulating leptin (*LEP*) expression and the YAP/TAZ-peroxisome proliferator-activated receptor gamma (PPARG) axis inhibiting PPARG target genes [406]. The WFS1/activating transcription factor 4 (ATF4)/zinc transporter 3 (ZNT3) signaling axis is implicated in palmitate-induced apoptosis, which is associated with obesity and depression, and the drug riluzole can target this axis to maintain zinc homeostasis [402]. Annexin A1 (ANXA1) influences the degradation of SMAD family member 4 (SMAD4) by competitively binding PDZ and LIM domain 7 (PDLIM7) against MYC binding protein 2 (MYCBP2), thereby downregulating *PPARG* transcription and inhibiting adipogenesis. ANXA1 deficiency exacerbates obesity and metabolic disorders (Fig. 7b). Additionally, increased expression of the histone acetyltransferase p300 is associated with ER stress and inhibits insulin signaling. The acetyltaurine hydrolase phosphotriesterase related (PTER) regulates food intake and resistance to obesity [407].

Single-cell sequencing holds significant potential in drug screening and gene therapy within obesity organoid models. In drug screening, analyzing single-cell data from organoid models can precisely identify drug targets involved in adipogenesis and the regulation of energy balance. For example, single-cell sequencing of adipose or brain organoids can reveal potential targets for treating obesity and its complications [408]. In evaluating the efficacy of gene therapy, the integration of CRISPR/Cas9 technology with single-cell sequencing presents novel avenues for optimizing treatment strategies and following gene editing in organoid models. For example, it can evaluate the effects of WFS1 gene editing on apoptosis and neural function in brain organoids, providing essential data for optimizing gene therapy strategies in obesity [408]. This includes evaluating impacts on neuronal apoptosis, metabolic function, and related gene expression networks. These data provide a crucial reference for designing personalized therapeutic approaches. Furthermore, establishing patient-specific organoid biobanks integrated with single-cell sequencing data facilitates the development of a molecular classification system for obesity, advancing precision medicine.

#### Hepatic steatosis

Non-alcoholic fatty liver disease (NAFLD) is a metabolic stress-induced liver injury closely associated with insulin resistance and genetic susceptibility [409]. Organoids offer innovative models for NAFLD. Human fetal liver cells have been used to construct organoids, with free fatty acids (FFAs) added to simulate steatosis induced by a Western diet. Additionally, the CRISPR/Cas9 system has been used to introduce the I148M mutation in patatin-like phospholipase domain containing 3 (PNPLA3) and knock out apolipoprotein B (*APOB*) and microsomal triglyceride transfer protein (*MTTP*), thereby creating organoid models with diverse genetic backgrounds [174]. Single-cell sequencing facilitates in-depth analysis of these organoid models. In a study on metabolic dysfunction-associated steatotic liver disease (MASLD), the new definition of NAFLD, organoid models exhibited varying cellular responses under different induction conditions (oleic acid, palmitic acid, and transforming growth factor beta 1 (TGF-β1). Single-cell sequencing revealed that all three models induced inflammatory signaling, but only TGF-β1 promoted collagen production, fibrosis, and hepatic stellate cell expansion. Oleic acid improved fibrosis characteristics and reduced the number of stellate cells. Correlating gene expression profiles related to MASLD progression demonstrated that palmitic acid and TGF-β1 more accurately mimic inflammation and fibrosis processes [410].

Dysregulation of lipid metabolism plays a crucial role in the pathogenesis of NAFLD. Fatty acid desaturase 2 (FADS2) is considered a key determinant of hepatic steatosis, as its increased expression raises polyunsaturated fatty acid levels and reduces de novo lipogenesis [411, 412]. The I148M mutation in PNPLA3 is closely associated with NAFLD, exacerbating steatosis in liver organoids and altering responses to FFA challenges [174]. Additionally, 26S proteasome regulatory subunit Rpn11 (*RPN11*) is upregulated in NAFLD, and its hepatocyte-specific knockout protects mice from diet-induced steatosis, insulin resistance, and hepatitis. RPN11 enhances N^6^-methyladenosine (m^6^A) modification of acyl-CoA synthetase short-chain family member 3 (*ACSS3*) by deubiquitinating and stabilizing methyltransferase 3 N^6^-adenosine-methyltransferase complex catalytic subunit (METTL3), thereby upregulating lipid-related genes [174]. During inflammation and fibrosis in NAFLD, factors including TNF activate inflammation, while TGF-β promotes hepatic stellate cell transdifferentiation into myofibroblasts, leading to extracellular matrix deposition and fibrosis (Fig. 7c). These processes drive NAFLD progression from lipid metabolism abnormalities to inflammation and fibrosis.

Combining single-cell sequencing with organoid models is highly valuable in NAFLD research. Analyzing gene expression in organoids can identify potential drug targets, FADS2, providing direction for novel drug development. Following gene editing in organoids, single-cell sequencing can assess the impact on cellular function and disease phenotype, evaluating changes after *PNPLA3* gene editing. This approach offers data support for developing gene therapy strategies in NAFLD [174].

Current liver organoids have limitations in simulating the complete hepatic microenvironment, notably lacking the involvement of immune cells, such as Kupffer cells, which may hinder a comprehensive understanding of inflammatory responses. In the field of gene therapy, single-cell sequencing can assess the effects of CRISPR/Cas9-mediated editing of the PNPLA3 gene on hepatic steatosis and inflammatory responses, providing a basis for personalized treatment[413]. Establishing a patient-specific organoid biobank, combined with single-cell sequencing data, enables the development of a molecular classification system for NAFLD, guiding precision treatment[174]. In summary, the powerful combination of organoid technology and single-cell sequencing has deepened our understanding of disease mechanisms and provided robust support for precision medicine approaches, including drug development and gene therapy.

The deep integration of single-cell sequencing and organoid technology has provided a powerful platform for systematically dissecting disease mechanisms in highly biomimetic in vitro models. Through in-depth research on four major categories of diseases, neurodegenerative diseases, genetic diseases, infectious diseases, and metabolic diseases, this technological combination enables precise identification of disease-associated rare cell subpopulations at single-cell resolution, delineation of lineage differentiation trajectories, and dynamic revelation of key signaling pathway abnormalities and complex intercellular interaction networks. With the continuous development of this technology in multi-omics integration, spatial analysis, and dynamic monitoring, it offers a novel perspective for fundamental disease biology research. It opens up a promising translational path for target discovery, drug screening, and the development of personalized treatment strategies.

## Single-cell sequencing for promoting tissue repair in organoids

Organ transplantation is an effective treatment for patients with organ damage or failure. However, ethical concerns, transplant rejection, organ supply scarcity, and the unique structure and function of the human brain significantly limit brain region transplantation [414]. Brain organoids derived from donors offer substantial cellular supply potential and share the host’s genetic background, reducing the risk of rejection. They represent an ideal source for regenerating and repairing diseased or damaged brain tissue [415].

Various types of organoids have been reported for transplantation, primarily in situ or ectopically, into mice, rats, or chick embryos, including kidney, intestinal, liver, pancreatic, brain, heart, lung, and retinal organoids, facilitating organoid growth and maintenance in vivo. Retinal organoid transplantation is an emerging therapeutic approach aimed at restoring vision in patients with visual loss. One study focused on using iPSC-derived photoreceptor cells for retinal transplantation to restore photoreceptive function in mice [416].

One study developed a technique using a specially prepared cholesterol mixture, primarily composed of cholesterol, to enable hiPSCs to generate functional brain organoids without the need for exogenous cells or genetic manipulation. These organoids were transplanted via specific routes into a liver fibrosis monkey model, successfully repairing damaged fibrotic liver tissue, thus offering a feasible pathway for advancing clinical research [417]. Another study demonstrated the in vitro culture of gallbladder-derived biliary epithelial cells into organoids, which were then transplanted into a mouse model of liver biliary disease, resulting in the repair of damaged intrahepatic bile ducts [418].

Currently, research on organoid transplantation and survival in vivo is primarily limited to animal models, with no reported studies on the safety and efficacy of organoids in humans. Some studies have demonstrated the transformation of iPSCs into endoderm stem cells (EnSCs) with endodermal differentiation specificity. Using EnSCs as seed cells, they have successfully engineered islet tissues (E-islets) in vitro for autologous regenerative islet transplantation therapy in patients. Postoperative results showed significant improvements in key indicators, fasting and postprandial C-peptide levels, confirming the effective restoration of islet function and indicating that such therapies might prevent the progression of diabetes complications [419].

The shortage of donor organs for transplantation remains a critical issue globally, and there is an urgent need for alternative therapies. Fetal cells and tissues have garnered attention for their substantial therapeutic potential. For example, organoid technology can utilize hiPSCs to generate unlimited quantities of human fetal-like cells and tissues [249]. Moreover, research has demonstrated that when hiPSC-derived liver organoids were transplanted onto the surface of host livers in a chemical fibrosis model, they recapitulated mid-gestational fetal liver characteristics, promoting liver regeneration and restoring liver function [420]. Additionally, the transplantation of biliary tree stem cell/early lineage-stage mesenchymal cell organoids onto the liver has shown that organoid patch grafts can protect hosts from genetically based disease states [421]. These studies highlight the vast potential of organoid technology in the field of alternative therapies, and future research and clinical trials will further advance its applications.

## Conclusions and future directions

The integration of single-cell sequencing with organoid technology is driving a fundamental paradigm shift in biomedical research [42, 422]. This synergistic technological innovation can provide notable insights into organ development and disease mechanisms through high-resolution analysis of cellular heterogeneity in organoids, dynamic tracking of lineage differentiation trajectories, and the elucidation of intercellular interaction networks [86, 423]. Compared to the technical limitations of traditional batch sequencing, which can only provide average signals across cell populations, single-cell sequencing has revolutionized our ability to decipher cellular heterogeneity [424]. It enables the precise identification of rare cell subpopulations and their unique transcriptional signature characteristics.

In colorectal cancer research, single-cell sequencing has revealed the abnormal activation of key signaling pathways, the Wnt-catenin beta 1 (CTNNB1/β-catenin) and phosphoinositide 3-kinase (PI3K)–protein kinase B (AKT) pathways [425]. More importantly, it has revealed the cell-type–specific roles of these pathways in the formation of tumor heterogeneity and the development of drug resistance. For instance, in breast cancer research, single-cell sequencing has uncovered key pathway networks driving tumor progression and therapeutic resistance, containing the Wnt–β-catenin, PI3K–AKT, and hypoxia-inducible factor pathways [426]. In lung cancer research, single-cell sequencing has identified the specific activation of the NOTCH signaling pathway in tumor-initiating cells and its central role in maintaining stem cell characteristics [427]. These signaling pathway maps resolved at a single-cell resolution provide a solid foundation for developing targeted therapies.

Additionally, in neurodegenerative disease research, brain organoid models can mimic key pathological features, amyloid β plaques and neurofibrillary tangles in Alzheimer’s disease, as well as the abnormal accumulation of synuclein alpha (SNCA/α-synuclein) in Parkinson’s disease, providing an ideal platform for studying their pathogenesis [271]. Notably, single-cell sequencing also enables in-depth analysis of cellular interaction networks within the tumor microenvironment, revealing dynamic interactions between cancer, immune, and stromal cells [428, 429]. These discoveries hold critical value for understanding disease progression mechanisms and optimizing therapeutic strategies.

Despite significant progress, this field still faces several critical technical bottlenecks. As 3D culture systems derived from stem cells or patient tissues, organoid models have become powerful tools for studying human diseases, but their practical applications remain fraught with multifaceted challenges [2]. In the construction of organoid models, standardization and reproducibility of culture systems remain prominent issues, with significant variations in culture protocols across laboratories substantially undermining the reliability of the results. In terms of model maturity, existing organoid models struggle to fully replicate the complex functions of adult organs, exhibiting critical functional deficiencies, incomplete metabolic enzyme systems, limited drug metabolism capacity, and immature electrophysiological properties [9, 430]. Their inadequacy in stimulating the microenvironment is particularly pronounced. Although breast cancer organoids can reproduce the histological and genetic heterogeneity of primary tumors, making them ideal models for studying subtype-specific behaviors and drug responses, the absence of functional immune cell populations, vascular networks, and innervation limits their application in investigating immune surveillance, blood–tissue barrier function, and neuro–immune axis interactions [431, 432]. Notably, advanced co-culture systems integrating organoids with immune or stromal cells are progressively overcoming these limitations, offering a more comprehensive understanding of how intercellular interactions shape disease progression.

Furthermore, high-throughput multi-omics experiments still face significant challenges in correcting batch effects, fusing multimodal data, and interpreting biological significance [433]. Single-cell sequencing also encounters analytical difficulties due to the complexity and high dimensionality of the data, including identifying cell dimers, detecting and filtering gene noise, and identifying rare cell populations [83, 434]. These challenges necessitate more robust bioinformatics tools and specialized expertise. Overcoming these technical bottlenecks will significantly advance the application of organoid models in both basic research and clinical translation. For instance, they will unlock greater value in fields including colorectal cancer research, where they have revealed how mutations in the APC regulator of the Wnt signaling pathway (*APC*) gene drive tumorigenesis through the Wnt signaling pathway [435–437].

To address these technical challenges, this review proposes the following innovative solutions and developmental directions. In the construction of engineered organoid models, novel biomaterials including stiffness-tunable hydrogels, functional peptide-modified extracellular matrix analogs, and organ-on-a-chip platforms based on microfluidic technology must be developed to achieve the precise construction of vascularized and immunized organoid models [438–440]. In terms of technological innovation, the integration of time-resolved single-cell multi-omics technologies with long-term live-cell imaging will drive a shift from static to dynamic profiling [441, 442]. In terms of computational methodology, there is a need to develop deep learning–based multi-omics data integration algorithms, cross-platform standardization tools, and causal inference models to better decipher gene regulatory networks and intercellular communication [442]. Notably, the integration of single-cell clustered regularly interspaced short palindromic repeats screening, spatial multi-omics, and organoid models will significantly enhance our ability to analyze gene functional networks and spatial organization structures in vitro systems [443].

From a translational medicine perspective, organoid technology is reshaping the landscape of drug development and clinical practice. Biobanks of organoids established from patient-specific iPSCs provide crucial resources for personalized medicine, bringing disease modeling and drug screening closer to clinical reality [444, 445]. In drug development, the integration of organoid models with single-cell sequencing can significantly enhance the predictive value of preclinical studies. For instance, combining high-throughput drug screening with single-cell sequencing can enable the simultaneous assessment of drug efficacy, toxicity, and cell type-specific responses. In precision medicine applications, this technology combination has yielded remarkable outcomes. For instance, in colorectal cancer research, the integration of single-cell sequencing with organoid models has revealed the pivotal role of the NLR family pyrin domain-containing 12 (NLRP12)–serine/threonine kinase 38 (STK38)–glycogen synthase kinase 3 beta (GSK3B) signaling axis in tumor development and progression [260, 446]. Additionally, in cystic fibrosis research, patient-derived intestinal and airway organoids were successfully employed to evaluate the modulator efficacy of the CF transmembrane conductance regulator (CFTR) and optimize personalized treatment strategies[447]. Moreover, in neuropsychiatric disorder research, brain organoid models have provided new insights into the cellular basis of autism spectrum disorders.

In summary, the integration of single-cell sequencing with organoid models will continue to drive innovative advancements in biomedical research. The further maturation of technologies, such as spatial transcriptomics, single-cell epigenomics, and single-cell proteomics, is expected to achieve the multidimensional analysis of entire organ development and disease progression. In terms of technological integration, emerging approaches such as artificial intelligence–driven multi-omics data mining, virtual organoid modeling, and digital twin technology will significantly enhance our understanding and prediction of complex biological processes. In terms of clinical translation, the integration of these two technologies will play an increasingly vital role in early disease diagnosis, the development of personalized treatment strategies, and regenerative medicine applications. However, realizing these visions will require sustained technological innovation and, more importantly, the establishment of interdisciplinary collaboration frameworks that will foster deep integration among biology, engineering, computational science, and clinical medicine, ultimately propelling precision medicine to higher levels of advancement and making significant contributions to human health.

## Data Availability

Not applicable.
